# Functional topologies of spatial cognition predict cognitive and motor progression in Parkinson’s

**DOI:** 10.3389/fnagi.2022.987225

**Published:** 2022-10-10

**Authors:** Deborah L. Harrington, Qian Shen, Xiangyu Wei, Irene Litvan, Mingxiong Huang, Roland R. Lee

**Affiliations:** ^1^Research Service, VA San Diego Healthcare System, San Diego, CA, United States; ^2^Department of Radiology, University of California, San Diego, La Jolla, CA, United States; ^3^Revelle College, University of California, San Diego, La Jolla, CA, United States; ^4^Department of Neurosciences, University of California, San Diego, La Jolla, CA, United States; ^5^Radiology Service, VA San Diego Healthcare System, San Diego, CA, United States

**Keywords:** Parkinson’s disease, spatial cognition, task-related functional connectivity, cognitive progression, motor progression, postural instability gait disturbances

## Abstract

**Background:**

Spatial cognition deteriorates in Parkinson’s disease (PD), but the neural substrates are not understood, despite the risk for future dementia. It is also unclear whether deteriorating spatial cognition relates to changes in other cognitive domains or contributes to motor dysfunction.

**Objective:**

This study aimed to identify functional connectivity abnormalities in cognitively normal PD (PDCN) in regions that support spatial cognition to determine their relationship to interfacing cognitive functions and motor disability, and to determine if they predict cognitive and motor progression 2 years later in a PDCN subsample.

**Methods:**

Sixty-three PDCN and 43 controls underwent functional MRI while judging whether pictures, rotated at various angles, depicted the left or right hand. The task activates systems that respond to increases in rotation angle, a proxy for visuospatial difficulty. Angle-modulated functional connectivity was analyzed for frontal cortex, posterior cortex, and basal ganglia regions.

**Results:**

Two aberrant connectivity patterns were found in PDCN, which were condensed into principal components that characterized the strength and topology of angle-modulated connectivity. One topology related to a marked failure to amplify frontal, posterior, and basal ganglia connectivity with other brain areas as visuospatial demands increased, unlike the control group (control features). Another topology related to functional reorganization whereby regional connectivity was strengthened with brain areas not recruited by the control group (PDCN features). Functional topologies correlated with diverse cognitive domains at baseline, underscoring their influences on spatial cognition. In PDCN, expression of topologies that were control features predicted greater cognitive progression longitudinally, suggesting inefficient communications within circuitry normally recruited to handle spatial demands. Conversely, stronger expression of topologies that were PDCN features predicted less longitudinal cognitive decline, suggesting functional reorganization was compensatory. Parieto-occipital topologies (control features) had different prognostic implications for longitudinal changes in motor disability. Expression of one topology predicted less motor decline, whereas expression of another predicted increased postural instability and gait disturbance (PIGD) feature severity. Concurrently, greater longitudinal decline in spatial cognition predicted greater motor and PIGD feature progression, suggesting deterioration in shared substrates.

**Conclusion:**

These novel discoveries elucidate functional mechanisms of visuospatial cognition in PDCN, which foreshadow future cognitive and motor disability.

## Introduction

Disturbances in spatial cognition are common in Parkinson’s disease (PD) on tests of spatial orientation and location, mental rotation, object and face recognition, visuospatial memory, and visuospatial construction (Weil et al., 2016; Oxtoby et al., 2021). These disturbances are a risk for future mild cognitive impairment (MCI) and dementia (Johnson and Galvin, 2011; Williams-Gray et al., 2013; Hong et al., 2014; Hobson and Meara, 2015; Chung et al., 2020; Ohdake et al., 2020). Visuospatial dysfunction can also contribute to motor disabilities, including freezing of gait (FoG) (Nantel et al., 2012; Chung et al., 2021), as walking depends upon the integration of spatial information in the environment with body-centered representations and cognitive-motor plans. However, the neuropathophysiology underlying spatial cognition has not been well delineated, especially before cognitive symptoms manifest. Markers that predate MCI and foreshadow the evolution of cognitive decline are vital as optimal treatments depend on early detection.

Despite an abundance of functional imaging studies in healthy adults (Cona and Scarpazza, 2019), functional changes in the brain during visuospatial cognition in PD have received little attention, especially in cognitively normal PD (PDCN). It is also unclear whether decline in spatial cognition relates to changes in other cognitive domains or contributes to motor dysfunction. The present study investigated the neural mechanisms of visuospatial cognition in PDCN and healthy adults who underwent functional MRI (fMRI) as they performed a mental rotation task. Participants judged whether a picture of a hand, rotated at various angles with respect to the body, depicted the left or right hand. The task elicits visuospatial imagery (Mibu et al., 2020; Nagashima et al., 2021) and implicit motor imagery (ter Horst et al., 2010). Brain activation typically increases with the amount of rotation performed, thereby serving as a proxy for visuospatial difficulty, rather than simple pattern recognition. The effects of visuospatial difficulty on brain activation are mixed in PD, with findings of decreased parietal (Nombela et al., 2014) and increased parietal-premotor cortex activation (Helmich et al., 2007). Discrepant results may be due to differences in paradigms and/or patients’ cognitive status. Moreover, activation intensity can be insensitive in disorders with heterogeneous neuropathophysiology such as PD, since regional activation may not be sufficiently or consistently altered across individuals (Rowe, 2010). Instead, task-modulated functional connectivity during a variety of cognitive tasks is often more sensitive to neuropathophysiology than regional activations, especially in patients without MCI (Harrington et al., 2014, 2018, 2020, 2021), and is essential for understanding interactions amongst multiple brain regions, which cognition and motor control depend upon.

To this end, our first aim was to identify disturbances in angle-modulated functional connectivity for frontal, posterior cortical and basal ganglia regions, which govern processes that support spatial cognition (Cona and Scarpazza, 2019). We hypothesized that connectivity of these regions with other brain areas would strengthen with visuospatial/visuomotor demands, but in a different manner in PDCN and healthy controls. The second aim was to identify relationships between mental rotation circuitry and cognitive functions that may support spatial cognition by correlating the strengths of abnormal functional connectivity topologies with performances in interfacing cognitive domains (e.g., working memory, executive, visuospatial memory). The third aim assessed the prognostic value of functional topologies by testing their ability to predict 2-year longitudinal changes in domain-specific cognition in a PDCN subsample. Aim four assessed the prognostic value of functional topologies, mental rotation proficiency, and domain-specific cognition in predicting longitudinal changes in motor symptoms, tremor, and postural instability and gait disturbance (PIGD) features. We hypothesized that declining spatial cognition would predict PIGD feature progression (Nantel et al., 2012; Chung et al., 2021).

## Materials and methods

### Participants and clinical assessments

The sample consisted of 63 PDCN who met the PD United Kingdom Brain Bank Criteria and 43 healthy controls. Exclusion criteria for all volunteers included metal in the head, neurological diagnoses other than PD, psychiatric diagnoses, history of alcohol or substance abuse, positive MRI findings, use of anticholinergics or cognitive medications, complaints of cognitive deficits, and severe tremor or dyskinesias that cause head motion. Volunteers were excluded if they met the Movement Disorders Society Level II criteria for PD-MCI (Litvan et al., 2012). MCI was defined as >1.5 standard deviations below the control group mean on at least two tests in single or different domains. There were six *de novo* patients, five patients taking dopamine agonist monotherapy, 26 taking levodopa monotherapy, and 26 taking levodopa combination therapy. All testing was conducted on medication therapy. Motor disability was assessed using the Movement Disorder Society Unified Parkinson’s Disease Rating Scale Part III (UPDRS Part III). Tremor and PIGD features were based on the sum of relevant items from the UPDRS Parts II and III (Stebbins et al., 2013). The Institutional Review Board at the VA San Diego Healthcare System approved the study. Subjects signed written informed consent prior to study procedures.

The groups did not differ in demographic characteristics (Table 1). A comprehensive battery that screened for MCI contained two tests for each of five domains (Table 1): attention and working memory (Color-Word Naming, CWN; Adaptive Digit Ordering, DOT); executive functioning (Letter Fluency, LET; Color-Word Interference, CWINH); visual and verbal memory (California Verbal Learning Test, CVLT; Brief Visuospatial Memory Test, BVMT); visual cognition as measured by visuospatial processing (Judgment of Line Orientation, JLOT) and visual organization (Hooper Visual Organization Test, HVOT); and semantic language as measured by confrontation naming (Boston Naming Test, BNT) and semantic fluency (Category Fluency, CAT). Two-years post-baseline testing, the UPDRS and alternate forms of the test battery were administered to a subsample of 41 PDCN, but not controls. All testing was conducted on medication therapy. No patient reported using anticholinergics or cognitive medications at the follow-up visit.

**TABLE 1:** Demographic, clinical, and cognitive characteristics of cohorts.

	Parkinson’s (*n* = 63)	Control (*n* = 43)	*P*	η_p_^2^
Age (years)	65.3 (6.5)	64.1 (8.5)	0.39	0.01
Education (years)	17.0 (2.1)	17.0 (2.1)	0.88	0.00
Sex (% females)	41.3%	44.2%	0.77	
Handedness (% right-handed)	84.1%	88.4%	0.54	
Wechsler test of adult reading^§^	44.4 (4.9)	45.6 (3.8)	0.22	0.02
Disease duration (years)	4.7 (3.8)			
Levodopa equivalent dosage^†^	927 (654)			
UPDRS^‡^ motor score	23.0 (11.4)			
UPDRS tremor score	4.5 (3.7)			
UPDRS PIGD score	1.7 (2.5)			
Hoehn and Yahr stage1:2:3:4	12:48:2:1			

**Head motion**				
Maximum rotation (degrees)	0.43 (0.26)	0.37 (0.20)	0.37	0.01
Maximum translation (mm)	0.58 (0.23)	0.54 (0.16)	0.37	0.01
Mean rotation (degrees)	0.12 (0.07)	0.11 (0.06)	0.38	0.01
Mean translation (mm)	0.20 (0.07)	0.19 (0.04)	0.24	0.01

**Attention and working memory**				
Adaptive digit ordering	6.4 (1.8)	6.6 (2.2)	0.58	0.00
DKEFS color + word naming	22.2 (7.3)	21.8 (4.5)	0.75	0.00

**Executive (DKEFS)**				
Letter fluency	45.2 (12.0)	49.3 (12.6)	0.09	0.03
Color-word interference	58.5 (12.9)	56.2 (11.3)	0.34	0.01

**Episodic memory**				
CVLT-II (long delay free recall)	9.1 (3.3)	11.3 (3.0)	0.001	0.11
BVMT-R (long delay free recall)	8.2 (2.6)	9.9 (1.9)	0.001	0.11

**Visual cognition**				
Judgment of line orientation	25.3 (2.8)	26.9 (2.7)	0.004	0.08
Hooper visual organization	25.4 (2.3)	27.3 (3.3)	0.001	0.10

**Semantic language**				
Boston naming	57.6 (2.6)	58.3 (1.7)	0.12	0.02
DKEFS category fluency	43.3 (8.7)	44.2 (9.1)	0.61	0.00

Tabled values are raw score means (standard deviations). Group differences were tested using ANOVA and Pearson chi-square statistics (sex, handedness). ^§^The Wechsler Test of Adult Reading is a measure of premorbid intelligence. ^†^Levodopa equivalent dosage was calculated using the method of Tomlinson (Tomlinson et al., 2010). Data are based on 57 participants who were taking dopaminergic therapy. ^‡^Movement Disorder Society Unified Parkinson’s Disease Rating Scale (UPDRS). The motor score is the sum of Part 3 item scores. Scores for tremor and postural instability gait disorder (PIGD) features are the sum of relevant items from Parts 2 and 3 (Stebbins et al., 2013). BVMT-R, Brief Visuospatial Memory Test-Revised; CVLT-II, California Verbal Learning Test Version 2; DKEFS, Delis Kaplan Executive Function System.

### Hand laterality task

Participants laid supine in the scanner with their arms at their sides, hands flat with palms down, and fingers pointing straight in the direction of the feet (i.e., 0° with respect to body). The left and right index fingers rested above a response key. Photographs of hands in the dorsal (back facing) position were oriented at 0°, 60°/300°, 120°/240°, and 180° with respect to the sagittal plane of the body (Supplementary Figure 1). Subjects judged whether the picture was a left or right hand by making a left or right index finger keypress. The 60°/300° and the 120°/240°conditions are equivalent rotation angles but are lateral (clockwise) and medial (counterclockwise) with respect to the body sagittal plane. Lateral and medial rotations minimized practice effects but were combined in the data analyses. The four rotation conditions were randomly presented eight times for each hand for a total of 64 trials. On each trial a picture was presented for 2,500 ms and the subject responded as quickly as possible. Intertrial intervals consisted of randomly jittered (1,000–3,900 ms) filler trials where the participant fixated on a central crosshair (Supplementary Figure 1). Randomized stimulus timing parameters were optimized using RSFgen from the Analysis of Functional NeuroImages (AFNI) 20.0 software.^1^ Task duration was 5 min 43 s. Outcomes were reaction time (RT) for correct trials (time from stimulus onset to keypress) and percent correct trials.

### MRI protocols

Imaging was conducted on a GE MR750 Discovery 3 Tesla system equipped with a Nova Medical 32-channel head coil. Visual stimuli were projected onto a screen and viewed through a mirror. Non-ferrous keypad devices recorded task performance. High-resolution T1-weighted images maximized differentiation of the gray and white matter boundary (3D spoiled gradient-recalled at steady state, minimum full TE, 3.5 ms; TR, 2,852 ms; TI, 1,000 ms; 8° flip angle; 0.8-mm slices, acquisition matrix = 512). Task-activated fMRI images used a high spatial and temporal resolution multiband-protocol (slice thickness = 2 mm, TR = 800 ms, TE = 35 ms, flip angle = 52°, acquisition matrix = 104, axial slices = 72, multiband factor = 8, echo spacing = 0.612 ms, band width = 4807.69 Hz/Px), which has greater sensitivity and specificity than single-band echo-planar protocols (Tomasi et al., 2016). The first 12.8 s were removed to allow magnetization to stabilize to a steady state. Total time of the fMRI run was 5 min and 57 s. A pair of gradient EPI sequences with anterior and posterior reversed gradients (TR = 8,500 ms; TE = 70.6 ms; isotropic voxels = 2 mm; flip angle = 90°; echo spacing = 0.612 ms) were acquired to correct geometric distortions.

### Image analyses

Functional images were preprocessed using FSL 6.4^2^ and AFNI. First a field map was computed from the pair of anterior and posterior reversed gradient sequences. Then it was applied to fMRI data to correct geometric distortions. Geometric distortion-corrected fMRI data were preprocessed using AFNI. Additional processing included (1) volume registration to the first echo-planar volume and head motion correction; (2) alignment to a skull-stripped T1-weighted structural image and warping to the MNI space; and (3) spatial smoothing using an isotropic Gaussian filter kernel with a full-width at half-maximum of 6 mm. There were no group differences in head motion (Table 1).

#### Voxelwise analyses of rotation angle and group effects

Analyses compared easy (0°, 60°/300°) and hard (120°/240°, 180°) angles of disparity conditions. AFNI 3dDeconvolve estimated the hemodynamic response function (HRF) of each voxel using multiple linear regressions. The pipeline included deconvolution of each subject’s time series for correct trials in each condition (easy/hard angles) and 12 motion parameters (six translational/rotational axes; six motion derivatives). Each HRF was estimated relative to the baseline state (filler images). Incorrect trials were regressed out of the time series at each voxel. Rotation angle effects were tested using a voxelwise probability of *p* < 0.0001 and a minimum cluster size of 19 voxels to obtain a familywise *p* < 0.05. The group and the group by rotation angle interaction effects were tested using a voxelwise of *p* < 0.001 and a minimum cluster size of 50 voxels to obtain a familywise *p* < 0.05.

#### Angle-modulated functional connectivity analysis (gPPI model)

Hypotheses focused on testing whether the strength of functional connectivity of a seed region of interest (ROI) with other brain areas as a function of rotation angle differed between the PDCN and control groups. To this end, the generalized psychophysical interaction (gPPI) model as implemented in AFNI was used. The gPPI approach analyses the physiological response of a ROI in terms of its angle-dependent coupling with other brain regions (McLaren et al., 2012). This produces measures of angle-modulated functional connectivity between two or more regions. Selection of seed ROI for the gPPI analyses was driven by regions known to play central roles in spatial cognition that also exhibited greater activation for hard than easy rotations in voxelwise analyses. Seeds (12 mm diameter) were placed in areas where peak activation was greater for hard than easy rotation angles. For basal ganglia nuclei, the seed encompassed all voxels. The physiological variable was created by extracting the mean deconvolved times-courses from a seed region for each subject. PPI interaction terms were computed as the cross product of the physiological variable and the angle condition. Nuisance variables were error trials for each angle condition and 12 motion regressors. This produced a first-level model with 14 nuisance variables and three regressors for each seed (time course of one seed, angle condition, interaction term). The resulting correlation maps for the time courses of seed ROI with the time courses from all other brain voxels as a function of rotation angle were then Fisher-z transformed. Second-level analyses tested the interaction of group with the angle contrast from the first-level analyses. A familywise *p* < 0.05 was obtained using a voxelwise probability of *p* < 0.005 and a minimum cluster size of 71 voxels for the cortex and 33 voxels for small-volume regions. The false discovery rate (FDR, *q* < 0.001) was applied to corrected *p*-values from the gPPI analyses to adjust for analyses of multiple seeds.

#### Brain atrophy

To determine if functional connectivity in PDCN was related to brain atrophy, cortical thickness and volume were analyzed using FreeSurfer 5.3^3^ (Supplementary material).

### Statistical analyses

#### Principal component analyses

Features that showed group differences in angle-modulated functional connectivity in the gPPI analyses were condensed into components using principal component analyses (PCA). Since the frontal cortex, posterior cortex, and the basal ganglia govern different facets of spatial cognition (Helmich et al., 2007; Weil et al., 2016; Garcia-Diaz et al., 2018), PCA was conducted separately for angle-modulated connectivity features of seeds within each of these areas to characterize their strength and topology of connectivity with the rest of the brain. An oblique rotation (Promax) implemented in SPSS 27 was applied. For each derived principal component (PC), a score was computed using the regression method, which converts variables into z scores, multiplies them by their pattern weight, and computes the weighted-linear combination of the variables. PC scores reflect the strength of regional angle-modulated couplings with other brain areas. PC scores were used in subsequent analyses to test for their correlations with behavioral variables.

#### Principal component score correlations with hand-laterality judgments

Stepwise multiple regression models entered sets of PC scores (frontal, posterior, basal ganglia seeds) as predictors of accuracy/RT (hard minus easy rotations), which were converted to age-adjusted residuals owing to associations of some variables with age, but not educational level. FDR correction was applied to uncorrected *p*-values (*q* ≤ 0.05).

#### Principal component score correlations with baseline and longitudinal cognitive decline

Stepwise regression models tested for sets of PC scores that correlated with baseline working memory, executive functions, visual episodic memory, visuospatial, and visual organization performances in each group. Cognitive variables were converted to age-adjusted residuals, as some variables correlated with age, but not educational level. In the PDCN subsample (*n* = 41), stepwise regressions tested for PC score predictors of longitudinal changes in all cognitive domains assessed by the study (Table 1). Longitudinal changes in age-adjusted residuals were calculated using simple discrepancy scores (score at baseline – score at visit 2). Uncorrected *p*-values from both baseline and longitudinal analyses were FDR corrected (*q* ≤ 0.05).

#### Predictors of baseline and longitudinal motor disability

Stepwise regressions tested for sets of PC scores that correlated with baseline motor, tremor, and PIGD feature severity (age-adjusted residuals). In the PDCN subsample, stepwise regressions tested whether PC scores, hand laterality measures, and longitudinal changes in cognition (age-adjusted simple discrepancy scores) predicted motor disability 2 years post-baseline and longitudinal motor progression (score at baseline – score at visit 2). Uncorrected *p*-values from both baseline and longitudinal analyses were FDR corrected (*q* ≤ 0.05).

## Results

### Hand-laterality judgments

**FIGURE 1:**
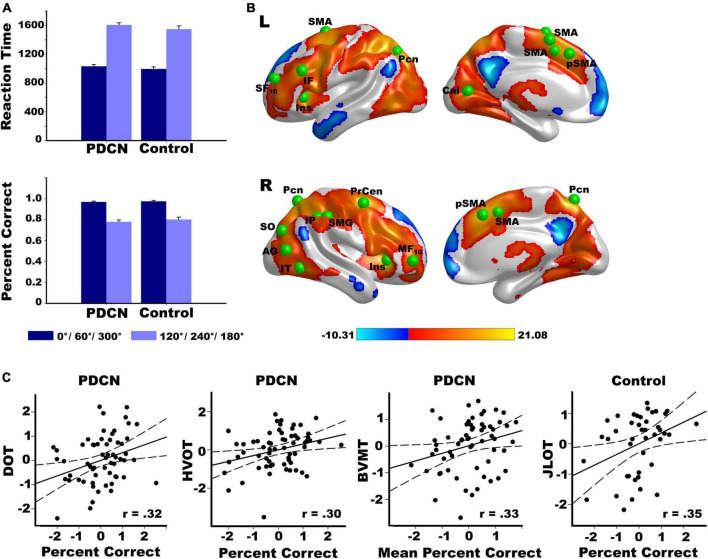
Rotation angle effects on hand laterality task performance and brain activation. **(A)** Larger angles of disparity with respect to the sagittal plane of the body (120°/240°/180°) were associated with longer reaction times and lower accuracy than smaller angles of disparity (0°/60°/300°) in both the PD and control groups. **(B)** Left and right hemisphere regional activations from voxelwise tests of rotation angle effects in all participants. Warm colors designate activations that were greater for larger (hard) than smaller (easy) angles of disparity. Cool colors designate activations that were greater for easy than hard angles of disparity. The color bar shows the range of *F*-values for significant angle effects. Green balls illustrate the locations of 20 cortical seeds of interest used in the gPPI analyses (Supplementary Table 1), which were placed in areas showing significantly greater activation for hard than easy rotation angles. The six basal ganglia seeds are not displayed (left/right caudate, putamen, globus pallidus. **(C)** Significant correlations between hand laterality accuracy and performance on tests of working memory (DOT), visual organization (HVOT), visuospatial cognition (JLOT), and visual episodic memory (BVMT) in the PD and control groups. Percent correct is indexed by accuracy for hard minus easy rotation angles. More negative percent correct values reflect lower accuracy for hard than easy rotation angles. Mean percent correct is average accuracy regardless of rotation angle. All measures are age-adjusted residuals. Brodmann areas for frontal seeds are designated by subscripts. AG, angular gyrus; Cal, calcarine cortex; IF, inferior frontal; IT, inferior temporal; Ins, anterior insula; IP, inferior parietal; IT, inferior temporal; MF, middle frontal; Pcn, precuneus; Precen, precentral; SF, superior frontal; pSMA, presupplementary motor area; SMA, supplementary motor area; SMG, supramarginal gyrus; SO, superior occipital; DOTA, Adaptive Digit Ordering Test (ascending order); BVMT, Brief Visuospatial Memory Test; JLOT, Judgment of Line Orientation Test; VOT, Hooper Visual Organization Test.

The analyses combined easy (0°, 60°/300°) and hard (120°/240°, 180°) rotation conditions to parallel the fMRI analyses. This classification was supported by analyses of all four rotation angles (Supplementary material and Supplementary Figure 2). The main effect of group and its interactions with angle and hand were non-significant for RT and percent correct. In both groups, RTs were longer [*F*(1,104) = 897, *p* < 0.0001, η_*p*_^2^ = 0.90] and accuracy was lower [*F*(1,104) = 189.9, *p* < 0.0001, η_*p*_^2^ = 0.65] for hard than easy rotations (Figure 1A). RTs were slower for the left than right hand (*F*(1,104) = 62.5, *p* < 0.0001, η_*p*_^2^ = 0.38), regardless of angle. Accuracy did not differ between hands. As rotation angle increased, performances were not influenced by upper-limb motor severity (UPDRS III) of the left or right hand (Supplementary material).

Regression analyses tested whether hand-laterality judgments (hard minus easy angle RT and accuracy; mean RT; mean accuracy) correlated with motor or cognitive variables. Performance did not correlate with motor, tremor, or PIGD feature severity. In PDCN, better working memory [DOT: (*F*(1,61) = 7.1, *p* < 0.01, *q* = 0.025, *r* = 0.32] and visual organization [HVOT: (*F*(1,61) = 6.1, *p* < 0.016, *q* = 0.037, *r* = 0.30] predicted greater accuracy for hard than easy rotations, and better visual memory [BVMT: *F*(1,61) = 7.5, *p* < 0.008, *q* = 0.012, *r* = 0.33] predicted higher accuracy for both angles (Figure 1C). In controls, better visuospatial processing correlated with better accuracy for difficult rotations [JLOT: (*F*(1,41) = 5.9, *p* < 0.02, *q* = 0.05, *r* = 0.35)].

### Group differences in brain morphometry

Group differences in cortical thickness and volume were non-significant (Supplementary material). Thus, gray matter was not used as a covariate in subsequent analyses.

### Voxelwise tests of brain activation

Figure 1B displays significant results from voxelwise tests of rotation angle effects on activation in all participants. Activation was typically greater for hard than easy rotation angles (warm colors) in both groups (Supplementary Figure 3). The voxelwise tests for the group and the group by rotation angle interaction effects on brain activation were non-significant (familywise *p* > 0.05 based on the voxelwise threshold of *p* < 0.001 and a minimum cluster size of 50 voxels).

### Group differences in angle-modulated connectivity (gPPI)

The study focused on testing whether angle-modulated functional connectivity of frontal, posterior, and basal ganglia areas differed between the groups. To this end, the gPPI model first identified significant angle-modulated functional connections of seed ROIs with other brain regions in all subjects, and then tested for group differences in these connections. Figure 1B and Supplementary Table 1 show 20 cortical seeds, which were placed in areas where peak activation was greater for hard than easy rotations. Twelve seeds were placed throughout frontal cortex and the anterior insula (hereafter referred to as frontal), which govern executive components of mental rotation (Cona and Scarpazza, 2019; Chung et al., 2020). Eight seeds were placed in parietal-occipital areas that support spatial cognition and action intentions (Zacks, 2008; Andersen and Cui, 2009), and temporal areas that support semantic or conceptual aspects of actions and the body (e.g., purpose, type such as lift or push, effortfulness, supramodal representations) (Binder and Desai, 2011; Lingnau and Downing, 2015; Brandman and Yovel, 2016). The six basal ganglia seeds (not shown) included bilateral caudate, putamen, and globus pallidus, which mediate cognitive-motor control during spatial cognition (Esposito et al., 2021). All voxels within each 12 mm diameter of cortical seeds and within basal ganglia seeds were activated for each subject.

The results showed that in one or both groups, hard rotation-angle seed time-courses correlated more strongly (positively) with the time courses of other brain voxels than easy rotation-angle seed time-courses. Supplementary Figure 4 and Supplementary Table 2 (Control > PDCN) and Supplementary Table 3 (PDCN > Control) describe the 53 features that showed group differences in the strength of angle-modulated connectivity (FDR adjusted, *q* < 0.001). No group differences were found for 85 other features that showed significant angle-modulated seed time courses (hard > easy) (Supplementary Figure 4 and Supplementary Table 4), indicating preservation of these connections in PDCN.

### Principal component analyses of abnormal angle-modulated connectivity features

**FIGURE 2:**
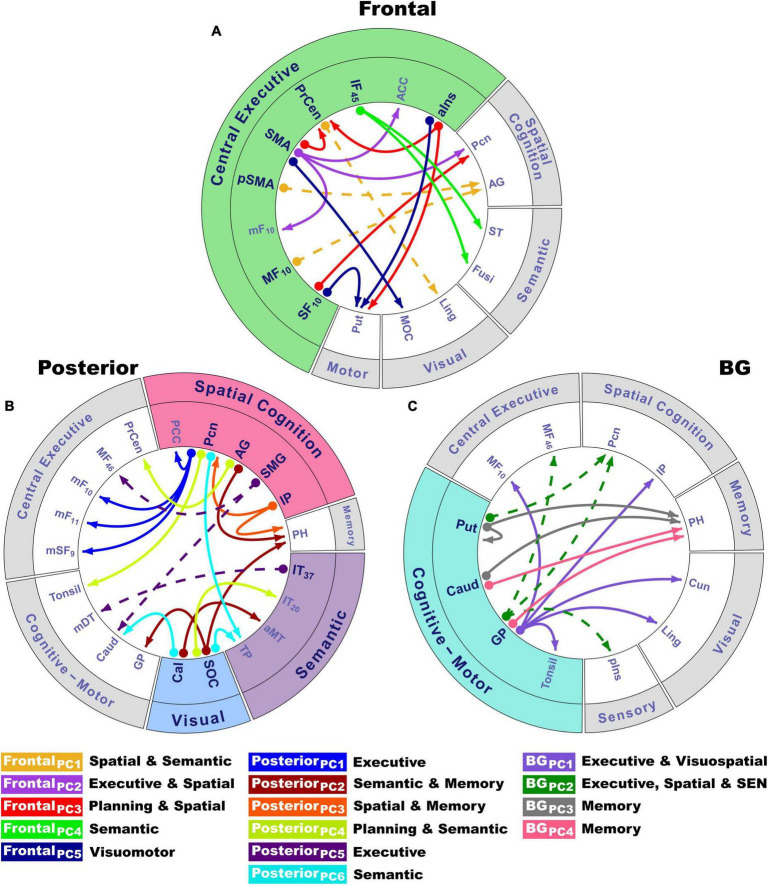
Circular visualization of principal component topologies for frontal, posterior and basal ganglia seeds. Principal components (PCs) are color-coded. Seed locations are bolded and designated by circles; arrows show the regions to which they connect. PC descriptions (bottom) broadly characterize the topologies of seed couplings with regions for which connectivity was stronger for difficult rotation angles. **(A)** Coupling topologies of frontal and anterior insula seeds involved in central executive functions (green rim). **(B)** Coupling topologies for posterior cortical seeds involved in visual (blue rim), spatial (pink rim), and semantic cognition (violet rim). **(C)** Coupling topologies of basal ganglia seeds, which modulate cognitive-motor control (turquoise rim). PC scores reflect the strength of angle-dependent seed couplings with other brain regions. Positive PC couplings (hard > easy angle connectivity) were features of either the control or the PDCN group (Table 2). Control features were positive PC couplings that were stronger in the control than in the PDCN group (designated by solid lines). PDCN features were positive PC couplings that were stronger in PDCN than in controls (designated by dashed lines). For each PC, the bottom caption broadly describes the cognitive function(s) of connecting regions with areas involved in executive and planning functions (frontal, precentral), spatial cognition (precuneus, angular gyrus, supramarginal gyrus, inferior parietal cortex), visual processes (occipital), semantic representations of actions and the body (temporal cortex), spatial memory (parahippocampus), and subcortical cognitive-motor control (basal ganglia, thalamus, cerebellum). PC topologies are detailed in Table 2. Numbers in subscripts are Brodmann areas. ACC, anterior cingulate; AG, angular gyrus; aIns, anterior insula; aMT, anterior middle temporal; BG, basal ganglia; Cal, calcarine cortex; Caud, caudate; Cun, cuneus; Fusi, fusiform gyrus; GP, globus pallidus; IF, inferior frontal; IP, inferior parietal; IT, inferior temporal; Ling, lingual gyrus; mDT, medial dorsal thalamus; mF, medial frontal; MF, middle frontal; MOC, middle occipital cortex; PCC, posterior cingulate cortex; Pcn, precuneus; PH, parahippocampus; pIns, posterior insula; PrCen, precentral; pSMA, presupplementary motor area; Put, putamen; SEN, somatosensory; SF, superior frontal; SMA, supplementary motor area; SMG, supramarginal gyrus; SOC, superior occipital cortex; ST, superior temporal; TP, temporal pole.

Due to the large number of group differences in angle-modulated connectivity features (53 features), PCA was used to reduce features into smaller sets of components for subsequent analyses testing for their associations with behavioral variables. PCA was performed separately for frontal, posterior, and basal ganglia seeds. PCs with eigenvalues ≥1.0 were extracted, resulting in five frontal, six posterior cortical, and four basal ganglia PCs, which, respectively accounted for 60, 65, and 59% of the cumulative variance. Each feature loaded on a single component (Table 2; matrix weightings ≥ ± 0.40). Figure 2 shows a circular visualization of the PCs. PC descriptions (Figure 2, bottom) broadly characterize the topologies of seed connections with areas involved in executive and planning functions, spatial cognition, visual processes, semantic representations of actions and the body, memory, and subcortical cognitive-motor control. PC scores reflect the strength of seed couplings with other brain regions as visuospatial demands increased. Table 2 shows that positive PC couplings (hard > easy angle connectivity) were features of either the control or the PDCN group. Positive PC couplings that were stronger in the control than in the PDCN group (Figure 2, solid lines) were designated as control features. Positive PC couplings that were stronger in PDCN than in controls (Figure 2, dashed lines) were designated as PDCN features. Multivariate ANOVAs showed significant group differences for frontal [*F*(5,100) = 62.3, *p* < 0.0001; η_*p*_^2^ = 0.76], posterior [*F*(6,99) = 52.1, *p* < 0.0001; η_*p*_^2^ = 0.76], and basal ganglia [*F*(4,101) = 44.6; *p* < 0.0001, η_*p*_^2^ = 0.64] PC scores. Group differences were associated with large effect sizes (Table 2; η_*p*_^2^ = 0.14 to.42). Disease duration and levodopa equivalent dosage did not significantly correlate with PC scores.

**TABLE 2:** Components characterizing group differences in angle-modulated coupling topologies.

Components/Seeds	Regional connectivity	Weights^†^	η_p_^2^

Central executive: Control > PDCN			
**Frontal PC 2**	**Executive and spatial**		**0.32**
LSMA	B medial frontal (BA 10), B anterior cingulate	0.89.86	
B SMA	L precuneus	0.57	
L SMA	R precuneus	0.54	

**Frontal PC 3**	**Planning and spatial**		**0.34**
R anterior insula	L precentral (BA 6), R putamen	0.85.45	
L superior frontal (BA 10)	B precuneus	0.72	
R SMA	L precentral (BA 6)	0.54	

**Frontal PC 4**	**Semantic**		**0.14**
L inferior frontal (BA 45)	L fusiform, R superior temporal	0.74.78	

**Frontal PC 5**	**Visuomotor**		**0.42**
R SMA	L middle occipital	–0.84	
L superior frontal (BA 10)	R putamen	0.75	
L anterior insula	R putamen	0.40	

**Central executive: PDCN > control**			
**Frontal PC 1**	**Visuospatial**		**0.28**
L preSMA	L angular gyrus, R angular gyrus	0.86.73	
R preSMA	L angular gyrus	0.77	
R middle frontal (BA 10)	L angular gyrus	0.53	
R precentral (BA 6)	L lingual gyrus	0.44	

**Visuospatial and semantic: Control > PDCN**			
**Posterior PC 1**	**Executive**		**0.24**
L precuneus	R medial superior frontal (BA 9),	0.77	
	L/R medial frontal (BA 11,10), R posterior cingulate	0.59.88.86	

**Posterior PC 2**	**Semantic and memory**		**0.40**
R superior occipital	L globus pallidus, LPH	0.71.79	
R angular gyrus	L PH	0.72	
L calcarine cortex	L anterior middle temporal	0.43	

**Posterior PC 3**	**Spatial and memory**		**0.30**
R inferior parietal	L/R precuneus, R PH	0.68.90.72	

**Posterior PC 4**	**Planning and semantic**		**0.40**
R angular gyrus	L precentral (BA 6)	–0.79	
R precuneus	R tonsil (lobule VIII)	0.65	
R superior occipital	R inferior temporal (BA 20)	–0.63	

**Posterior PC 6**	**Semantic**		**0.09**
L calcarine cortex	R caudate	0.63	
L precuneus	L temporal pole	0.54	
R superior occipital	L temporal pole	0.48	

**Visuospatial and semantic: PDCN > control**			
**Posterior PC 5**	**Executive**		**0.26**
R SMG	R middle frontal (BA 46), R caudate	0.78.71	
R inferior temporal (BA 37)	B medial dorsal thalamus	0.48	

**Cognitive-motor control: Control > PDCN**			
**Basal ganglia PC 1**	**Executive and visuospatial**		**0.24**
L globus pallidus	L tonsil (lobule VIIIa), R cuneus, L lingual gyrus	0.76.81.78	
	R middle frontal (BA 10), R inferior parietal	0.55.71	

**Basal ganglia PC 3**	**Memory**		**0.32**
L caudate	L PH	0.70	
L putamen	R PH, R putamen	–0.66.40	

**Basal ganglia PC 4**	**Memory**		**0.23**
L caudate	R PH	0.82	
R globus palllidus	R PH	0.64	

**Cognitive-motor control: PDCN > control**			
**Basal ganglia PC 2**	**Executive, spatial and somatosensory**		**0.30**
L globus pallidus	L precuneus/superior parietal, middle frontal	0.87.86	
L putamen	L posterior insula, L precuneus	0.73.47	

^†^Pattern matrix weights for seed connections. Group test effect sizes (η_p_^2^). BA, Brodmann area; B, bilateral; L, left; R, right; PH, parahippocampus; SMA, supplementary motor area; SMG, supramarginal gyrus.

### Principal component score correlations with hand-laterality judgments

Stepwise regressions tested for PC scores that best predicted task performance (age-adjusted residuals) (FDR corrected). In controls, but not PDCN, longer RTs correlated with stronger frontal PC_1_ scores [*F*(1,41) = 5.0, *p* < 0.02, *q* = 0.03, *r* = 0.33] (PD feature); stronger posterior PC_4_ (r_*xy.z*_ = 0.50), PC_6_ (r_*xy.z*_ = 0.46), and PC_1_ scores (r_*xy.z*_ = 0.35) [*F*(3, 39) = 8.9, *p* < 0.0001, *q* = 0.016, *R* = 0.64] (control features); and stronger basal ganglia PC_4_ scores (control feature) predicted lower accuracy [*F*(1,41) = 4.6, *p* < 0.039, *q* = 0.05, *r* = −0.32] (Supplementary Figure 5).

### Principal component score correlations with baseline cognition

**FIGURE 3:**
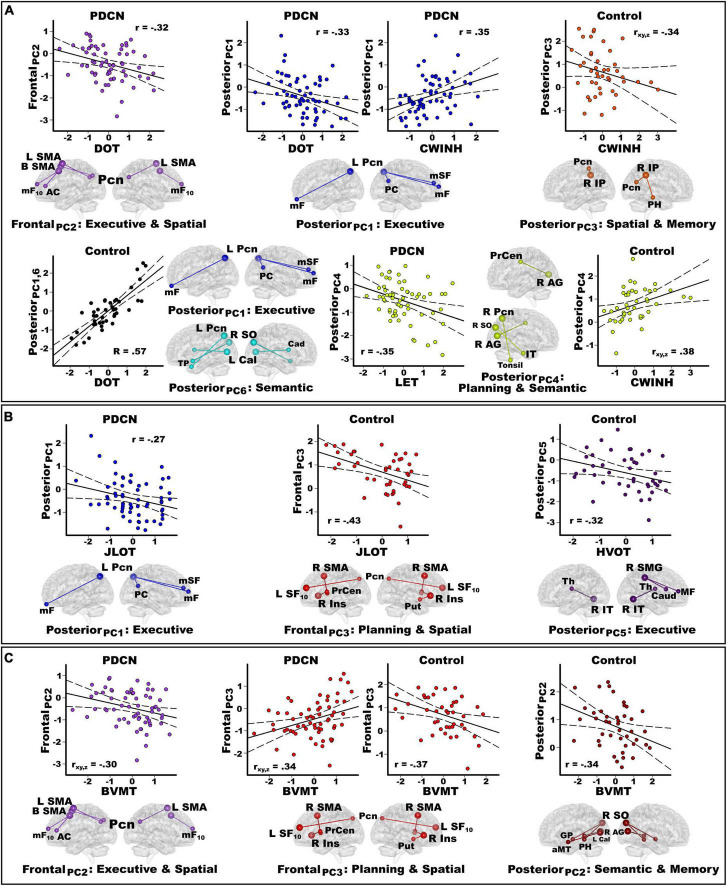
Principal component score correlations with baseline cognition in the PDCN and control groups. Plots show significant results from stepwise multiple regressions testing for sets of PC scores (frontal, posterior, basal ganglia) that best correlated with baseline working memory and executive functions **(A)**, spatial cognition **(B)**, and visual episodic memory **(C)** performances in each group. Plots display the best-fitting linear regression line (solid line) and 95% conference intervals (dotted lines) for significant correlations between age-adjusted cognitive measures (*x*-axis) and PC connectivity topologies (*y*-axis). Higher cognitive scores reflect better performance except for the CWINH where higher scores signify poorer performance. For each PC, seed(s) (large balls) and their connection(s) (small balls) are color-coded. **(A)** Working memory (DOT) and executive functions (CWINH, LET) significantly correlated with frontal and posterior PC scores in the PD group and posterior PCs in the control group. In the control group, the regression equation contained two predictors (posterior PC_1_ and PC_6_) that both correlated positively with DOT performances. Thus, predicted values from the regression model are plotted for posterior PC_1_ and PC_6_ [Σ intercept + (beta_PC1_ * PC_1_ score) + (beta_PC6_ * PC_6_ score)] = [Σ–0.39 + (0.44 * PC_1_ score) + (0.36 * PC_6_ score)]. The associated R value is the correlation with both predictors in the equation. In controls, posterior PC_3_ and PC_4_ scores both predicted CWINH performances (*R* = 0.48) but correlated negatively and positively. Partial correlations (r_xy_._z_) are plotted for each predictor to show their relationship with CWINH scores after adjusting for effects of the other predictor variable. **(B)** Spatial cognition (JLOT, HVOT) significantly correlated with a posterior PC score in PDCN and frontal and posterior PC scores in the control group. **(C)** Visual memory (BVMT) correlated with frontal PC scores in the PD group and frontal and posterior PC scores in controls. In the PDCN group, frontal PC_2_ and PC_3_ both predicted BVMT performances (*R* = 0.44) but correlated negatively and positively. Partial correlations (r_xy_._z_) are plotted for each predictor to show their relationship with BVMT scores after adjusting for effects of the other predictor variable. Brodmann areas for frontal seeds are designated by subscripts. L, left hemisphere; R, right hemisphere; B, bilateral hemispheres. AC, anterior cingulate; AG, angular gyrus; Cad, caudate; Cal, calcarine cortex; Ins, insula; IP, inferior parietal; IT, inferior temporal; mF, medial frontal; mSF, medial superior frontal; mF, medial frontal; mSF, medial superior frontal; MF, middle frontal; Pcn, precuneus; PC, posterior cingulate; PH, parahippocampus; PrCen, precentral; Put, putamen; SF, superior frontal; SO, superior occipital; SMA, supplementary motor area; Thal, thalamus; TP, temporal pole. DOT, Adaptive Digit Ordering Test; BVMT, Brief Visuospatial Memory Test; CWINH, Color-Word Inhibition test; JLOT, Judgment of Line Orientation Test; LET, Verbal Fluency Letters; HVOT, Hooper Visual Organization Test.

Table 1 shows that at baseline the PDCN group performed worse than controls on episodic memory and spatial cognition. No group differences were found on the remaining neuropsychological tests. Figure 3 displays the results from stepwise regressions testing for sets of PC scores that best correlated with neuropsychological functions that interface with spatial cognition (FDR corrected).

#### Working memory (DOT)

Figure 3A shows that in PDCN, stronger frontal PC_2_ [*F*(1,61) = 6.9, *p* < 0.01, *q* = 0.028, *r* = −0.32] and stronger posterior PC_1_ scores [*F*(1,61) = 7.5, *p* < 0.008, *q* = 0.025, *r* = −0.33] (control features) predicted poorer working memory. In contrast, stronger posterior PC_1_ (r_*xy.z*_ = 0.46) and posterior PC_6_ scores (r_*xy.z*_ = 0.40) (control features), predicted better working memory in controls [*F*(2,40) = 9.7, *p* < 0.0001, *q* = 0.003, *R* = 0.57].

#### Executive functioning (CWINH, LET)

Figure 3A shows that in PDCN, stronger posterior PC_1_ scores (control feature) predicted poorer inhibition [*F*(1,60) = 8.5, *p* < 0.005, *q* = 0.018, *r* = 0.35; one outlier removed]. In controls, stronger posterior PC_3_ (r_*xy.z*_ = −0.34) and posterior PC_4_ scores (r_*xy.z*_ = 0.38) (control features), respectively predicted better and poorer inhibition [*F*(2,40) = 6.1, *p* < 0.005, *q* = 0.015, *R* = 0.48]. In PDCN only, strong posterior PC_4_ scores predicted poorer phonemic fluency [*F*(1,61) = 8.3, *p* < 0.005, *q* = 0.013, *r* = −0.35].

#### Spatial cognition (JLOT, HVOT)

Figure 3B shows that in PD, stronger posterior PC_1_ scores (control feature) correlated with poorer visuospatial cognition [*F*(1,61) = 4.8, *p* < 0.03, *q* = 0.04, *r* = −0.27]. In controls, stronger frontal PC_3_ scores (control feature) correlated poorer visuospatial cognition [*F*(1,41) = 9.5, *p* < 0.004, *q* = 0.01, *r* = −0.43)], whereas stronger posterior PC_5_ scores (PD feature) correlated with poorer visual organization [*F*(1,41) = 4.5, *p* < 0.04, *q* = 0.045, *r* = −0.32].

#### Visuospatial memory (BVMT)

Figure 3C shows that in PD, stronger frontal PC_3_ (r_*xy.z*_ = 0.34) and frontal PC_2_ scores (r_*xy.z*_ = −0.30) (control features), respectively correlated with better and worse visual memory [*F*(2,60) = 5.8, *p* < 0.002, *q* = 0.005, *R* = 0.44)]. In controls, stronger frontal PC_3_ correlated with poorer visual memory [*F*(1,41) = 6.6, *p* < 0.015, *q* = 0.03, *r* = −0.37], whereas stronger posterior PC_2_ scores (control feature) correlated with poorer visual memory [*F*(1,41) = 5.4, *p* < 0.025, *q* = 0.038, *r* = −0.34].

### Principal component score predictors of longitudinal cognitive decline

**FIGURE 4:**
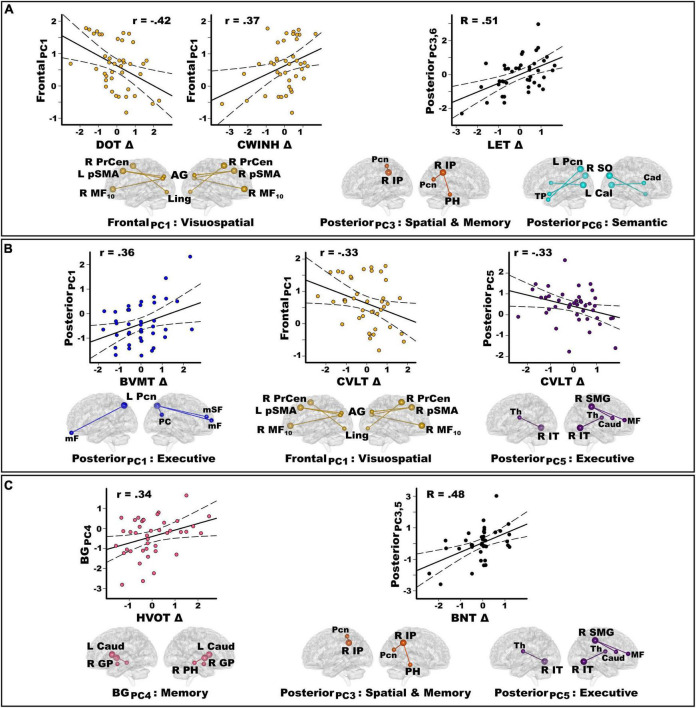
Principal component score predictors of longitudinal changes in cognition in the PDCN subsample. Stepwise multiple regressions tested for sets of PC scores (frontal, posterior, basal ganglia) that best predicted longitudinal changes in each cognitive domain assessed by the study. Plots display the best-fitting linear regression line (solid line) and 95% conference intervals (dotted lines) for PC predictors (*y*-axis) of longitudinal changes in cognition (*x*-axis) as measured by age-adjusted simple discrepancy scores (Δ) (score at baseline – score at visit 2). Positive discrepancy scores signify cognitive decline at the follow-up visit, except for the CWINH where negative discrepancy scores signify cognitive decline. For each PC, seed(s) (large balls) and their connection(s) (small balls) are color-coded. **(A)** Frontal and posterior PC scores significantly predicted longitudinal changes in working memory (DOT) and executive functions (CWINH, LET). The regression equation contained two predictors (posterior PC_3_ and PC_6_) that both correlated positively with longitudinal changes in LET scores. Thus, predicted values from the regression model are plotted for posterior PC_3_ and PC_6_ [Σ intercept + (beta_PC3_ * PC_3_ score) + (beta_PC6_ * PC_6_ score)] = [Σ0.36 + (0.58 * PC_3_ score) + (0.39 * PC_6_ score)]. The R value is the correlation with both predictors in the equation. **(B)** Frontal and posterior PC scores significantly predicted longitudinal changes in visual (BVMT) and verbal episodic memory (CVLT). **(C)** Posterior and basal ganglia PC scores predicted longitudinal changes in visual organization (HVOT) and confrontation naming (BNT). The regression equation contained two predictors (posterior PC_3_ and PC_5_) that both correlated positively with longitudinal changes in BNT scores (*R* = 0.48). Predicted values from the regression model are plotted for posterior PC_3_ and PC_5_ [Σ intercept + (beta_PC3_ * PC_3_ score) + (beta_PC5_ * PC_5_ score)] = [Σ–0.18 + (–0.42 * PC_3_ score) + (–0.30 * PC_5_ score)]. Brodmann areas for frontal seeds are designated by subscripts. L, left hemisphere; R, right hemisphere; AG, angular gyrus; Cad, caudate; Cal, calcarine cortex; GP, globus pallidus; IP, inferior parietal; IT, inferior temporal; Ling, lingual gyrus; mF, medial frontal; mSF, medial superior frontal; MF, middle frontal; Pcn, precuneus; PC, posterior cingulate; PH, parahippocampus; PrCen, precentral; pSMA, presupplementary motor area; SO, superior occipital; SMG, supramarginal gyrus; Thal, thalamus; TP, temporal pole. DOT, Adaptive Digit Ordering Test; BNT, Boston Naming Test; BVMT, Brief Visuospatial Memory Test; CWINH, Color-Word Inhibition test; CVLT, California Verbal Learning Test; LET, Verbal Fluency Letters; HVOT, Hooper Visual Organization Test.

Forty-one PDCN completed neuropsychological testing 2-years post-baseline (range = 19.9–32.8 months) (Table 3). The number of months between testing did not correlate with longitudinal cognitive changes. At follow-up, no patient had a clinical diagnosis of dementia. Nine patients (22%) exhibited MCI (>−1.5 SD on two or more tests), with eight showing multidomain and one showing single-domain MCI. Longitudinal decline in the PDCN subsample was significant for executive functions, visual cognition, and semantic fluency (Table 3). Figure 4 displays the results from stepwise regressions (FDR adjusted) testing for sets of PC scores that best predicted longitudinal changes in cognition.

**TABLE 3:** Demographic, clinical, and cognitive characteristics of the PDCN subsample.

Age (years)	64.5 (7.0)			
Education (years)	17.0 (2.2)			
Sex (% females)	39%			
Disease duration (years)	4.3 (3.4)			
Months between baseline and follow up	24.6 (3.2)			

	**Visit 1**	**Visit 2**	**p**	**d^±^**
UPDRS^‡^ motor score	24.8 (12.5)	29.1 (14.7)	0.001	0.63
UPDRS tremor score	4.4 (3.5)	3.7 (3.2)	0.11	0.25
UPDRS PIGD score	1.9 (2.6)	2.9 (3.1)	0.001	0.62
Levodopa equivalent dosage^†^	816.7 (669.2)	1239.9 (789.7)	0.001	0.79

**Attention and working memory**				
Adaptive digit ordering	6.7 (1.9)	6.1 (2.7)	0.045	0.27
DKEFS Color + word naming	23.0 (8.4)	21.0 (4.3)	0.09	0.27

**Executive (DKEFS)**				
Color-word interference	58.6 (14.4)	63.5 (22.5)	0.009	0.39
Phonemic fluency (letters)	47.2 (11.7)	43.0 (11.4)	0.001	0.55

**Episodic memory (long delay free recall)**				
CVLT (long delay free recall)	9.4 (3.3)	8.9 (5.1)	0.17	0.15
BVMT (long delay free recall)	8.6 (2.9)	8.6 (2.9)	0.50	0.00

**Visual cognition**				
Judgment of line orientation	25.3 (2.7)	24.1 (4.6)	0.02	0.33
Hooper visual organization	25.3 (2.8)	23.6 (3.9)	0.001	0.58

**Semantic language**				
Boston naming	57.9 (2.6)	57.9 (3.0)	0.43	0.03
DKEFS category fluency	44.4 (9.1)	39.0 (9.2)	0.001	0.62

Tabled values are raw score means (standard deviations) from a subsample of 41 PDCN participants. Longitudinal changes between baseline (Visit 1) and follow-up (Visit 2) testing were analyzed using paired t-tests with bias-corrected accelerated bootstrapping (1,000 iterations). Longitudinal changes in age-adjusted residuals (Figure 4) were calculated from these data using simple discrepancy scores (age-adjusted score at baseline – age-adjusted score at visit 2). ^‡^Movement Disorder Society Unified Parkinson’s Disease Rating Scale (UPDRS). The motor score is the sum of Part 3 item scores. Scores for tremor and postural instability gait disorder (PIGD) features are the sum of relevant items from Parts 2 and 3 (Stebbins et al., 2013). ± Cohen’s d. ^†^Levodopa equivalent dosage was calculated using the Tomlinson method (Tomlinson et al., 2010). Data are based on 36 participants who were taking dopaminergic medications at both baseline and follow-up testing. BVMT-R, Brief Visuospatial Memory Test-Revised; CVLT, California Verbal Learning Test Version 2; DKEFS, Delis Kaplan Executive Function System.

Figure 4A shows that stronger frontal PC_1_ scores (PD feature) predicted less decline or preserved working memory [*F*(1,39) = 8.4, *p* < 0.006, *q* = 0.02, *r* = −0.42] and response inhibition [*F*(1,39) = 6.0, *p* < 0.019, *q* = 0.03, *r* = 0.37]. Stronger posterior PC_3_ (r_*xy.z*_ = 0.43) and posterior PC_6_ (r_*xy.z*_ = 0.39) scores (control features) predicted greater decline in phonemic fluency [*F*(2,38) = 6.6, *p* < 0.003, *q* = 0.007, *R* = 0.51]. Figure 4B shows that stronger posterior PC_1_ scores (control feature) predicted greater decline in visuospatial memory [*F*(1,39) = 5.8, *p* < 0.02, *q* = 0.035, *r* = 0.36], whereas stronger frontal PC_1_ scores and posterior PC_5_ scores (PD feature) both predicted less decline or preserved verbal memory [frontal PC_1_: *F*(1,39) = 4.7, *p* < 0.036, *q* = 0.047, *r* = −0.33; posterior PC_5_: *F*(1,39) = 4.7, *p* < 0.036, *q* = 0.045, *r* = −0.33]. Figure 4C shows that stronger basal ganglia PC_4_ scores (control feature) predicted greater decline in visual organization [*F*(1,39) = 5.1, *p* < 0.03, *q* = 0.04, *r* = 0.34]. Stronger posterior PC_3_ scores (r_*xy.z*_ = −0.40) (control feature) and posterior PC_5_ scores (r_*xy.z*_ = −0.33) (PD feature) predicted greater decline in confrontation naming [*F*(2,37) = 5.7, *p* < 0.007, *q* = 0.02, *R* = 0.48; one outlier removed].

### Predictors of baseline and longitudinal motor disability

**FIGURE 5:**
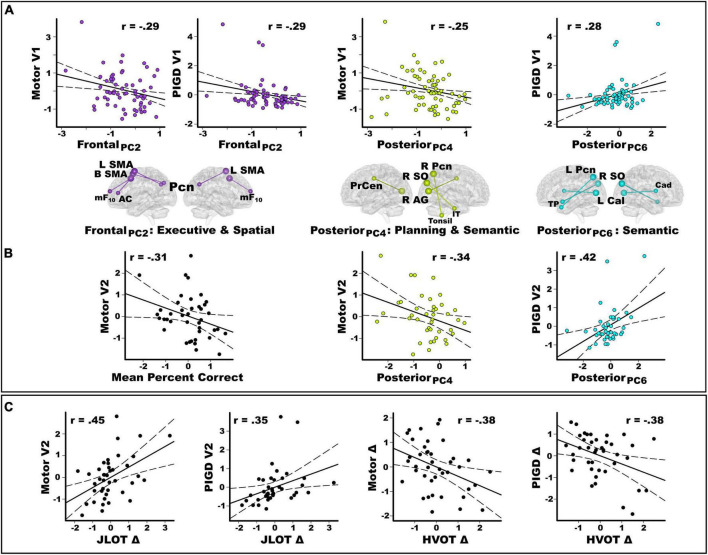
Predictors of baseline and longitudinal changes in motor disability. Plots display the best-fitting linear regression line (solid line) and 95% conference intervals (dotted lines) for predictors (*x*-axis) of motor severity (*y*-axis). **(A)** Stepwise regressions tested for sets of PC scores (frontal, posterior, basal ganglia) that correlated with baseline motor disability in the entire PDCN sample (*n* = 63). There were significant frontal and posterior PC score predictors of baseline motor severity (UPDRS III) and PIGD feature severity at the baseline study visit (V1). **(B)** In the PDCN subsample (*n* = 41), stepwise regressions tested whether mental rotation accuracy and sets of PC scores (frontal, posterior, basal ganglia) predicted motor disability scores 2 years post-baseline. Graphs show that mental rotation accuracy predicted motor severity at the second study visit (V2). Posterior PC scores predicted the motor and PIGD feature severity at the second study visit. **(C)** In the PDCN subsample, stepwise regressions tested whether longitudinal changes in spatial cognition predicted motor disability scores at the second study visit (V2) or longitudinal changes in motor disability (score at baseline – score at visit 2). Greater longitudinal decline (Δ) in visuospatial cognition (JLOT) predicted greater motor and PIGD feature severity 2 years post-baseline (V2). Greater longitudinal decline in visual organization (HVOT) predicted greater longitudinal increases (Δ) in motor and PIGD feature severity. For motor variables, negative simple discrepancy scores (score at baseline visit 1 – score at visit 2) signify greater motor symptoms at follow-up than baseline testing. For cognitive variables, positive simple discrepancy scores signify cognitive decline at the follow-up visit relative to baseline. All cognitive and motor measures are age-adjusted residuals. HVOT, Hooper Visual Organization Test; JLOT, Judgment of Line Orientation Test; PIGD, postural instability gait disorder; UPDRS, Movement Disorders Society Unified Parkinson’s Disease Rating Scale.

Stepwise regressions tested for sets of PC scores that best explained baseline motor disability. Figure 5A shows that stronger frontal PC_2_ scores (control feature) correlated with lower motor [*F*(1,61) = 5.5, *p* < 0.02, *q* = 0.027, *r* = −0.29] and PIGD feature severity [*F*(1,61) = 5.5, *p* < 0.02, *q* = 0.03, *r* = −0.29]. Stronger posterior PC_4_ scores (control feature) correlated with lower motor severity [*F*(1,61) = 4.0, *p* < 0.049, *q* = 0.05, *r* = −0.25]. Stronger posterior PC_6_ scores (control feature) correlated with greater PIGD feature severity [*F*(1,61) = 5.3, *p* < 0.024, *q* = 0.036, *r* = 0.28]. PC scores did not correlate with tremor severity.

In the PDCN subsample, the number of months between UPDRS testing did not correlate with longitudinal changes in motor disability. Motor and PIGD feature severity, but not tremor severity, significantly increased longitudinally (Table 3). Stepwise regressions tested for hand laterality, PC scores, and cognitive predictors of (1) motor disability 2 years post-baseline (age-adjusted residuals) and (2) symptom progression (age-adjusted discrepancy scores: score at baseline – score at visit 2). Figure 5B shows that poorer hand-laterality accuracy for both angles predicted greater motor severity 2 years post-baseline [*F*(1,39) = 4.3, *p* = 0.045, *q* = 0.046, *r* = −0.31]. Stronger posterior PC_4_ and PC_6_ scores (control features), respectively predicted lower motor severity [*F*(1,39) = 5.1, *p* = 0.03, *q* = 0.04, *r* = −0.34] and greater PIGD feature severity [*F*(1,39) = 8.4, *p* < 0.006, *q* = 0.009, *r* = 0.42] at follow-up. PC scores did not predict longitudinal changes in tremor severity.

Figure 5C shows that greater longitudinal decline in visuospatial cognition (positive discrepancy scores) predicted greater motor [*F*(1,39) = 10.0, *p* = 0.003, *q* = 0.005, *r* = 0.45] and PIGD feature severity [*F*(1,39) = 5.5, *p* = 0.02, *q* = 0.023, *r* = 0.35] at follow-up. Greater longitudinal decline in visual organization predicted greater longitudinal progression of motor [*F*(1,39) = 6.7, *p* < 0.014, *q* = 0.015, *r* = −0.38] and PIGD features [*F*(1,39) = 6.6, *p* < 0.014, *q* = 0.014, *r* = −0.38]. Cognitive variables were not associated with longitudinal changes in tremor severity.

## Discussion

Spatial cognition impairments are common in PD, but scant attention has been paid to the functional mechanisms especially in lifelike contexts where adjustments to fluctuating visuospatial demands are called for. Despite the absence of group differences in regional activation, we found that regional functional connectivity was amplified with other brain areas as rotation angle increased, largely in elderly controls who flexibly engaged cognitive resources in accord with mental rotation demands. This result underscores the importance of leveraging complementary functional connectivity approaches to understand the neuropathophysiology of cognition in PDCN, which are sensitive to communications amongst multiple brain regions (Harrington et al., 2014, 2018, 2020, 2021). In this regard, we discovered two aberrant connectivity patterns in PDCN, which were condensed into principal components that characterized the strength and topology of regional couplings to increasing spatial demands. One topology related to a marked failure to amplify frontal, posterior, and basal ganglia communications with other brain areas as spatial demands increased, unlike the control group (control features). Another topology was related to functional reorganization, whereby connectivity of distinct brain areas was amplified with regions not recruited by the control group (PDCN features). Coupling topologies correlated with different facets of cognition, albeit differently between groups, underscoring the influence of interfacing processes on spatial cognition. The expression of PDCN and control group topologies had different prognostic implications for longitudinal cognitive progression, suggesting distinct underlying mechanisms. Parieto-occipital coupling topologies and longitudinal decline in spatial cognition were both prognostic of motor and PIGD feature severity 2 years later.

### Coupling topologies correlate with baseline cognition

PDCN patients exploited multiple cognitive resources to support mental transformations as better hand-laterality accuracy correlated with better working memory, visual organization, and visual memory (Scarpina et al., 2019). Controls, however, mainly enlisted visuospatial resources during mental rotation as accuracy correlated only with visuospatial processing ability. In turn, domain-specific cognitive processes also correlated with mental-rotation evoked coupling topologies, but differently in each group. Some topologies predicted performances on several neuropsychological tests, as general processing resources are shared across different cognitive functions (Salthouse, 2017; Tucker-Drob et al., 2019). Poorer cognition in PDCN typically correlated with stronger expression of frontal and posterior topologies that were control group features. These results suggest inefficiencies in long-range communications of diverse posterior (PC_1_,_2_,_3_,_4_,_6_) and frontal topologies, including the SMA (PC_2_,_5_), which is dysfunctional in PD (Herz et al., 2014). These findings may therefore signify emerging neuropathophysiology in brain circuits that healthy controls flexibly enlist to handle increasing visuospatial demands. This interpretation aligns with the finding that greater longitudinal cognitive decline in PDCN was predicted by stronger expression of control topologies at baseline (see below). Conversely, in the control group stronger expression of control topologies typically correlated with poorer cognition, which is compatible with the greater recruitment of neuronal resources as task demands increase, especially in elders who are poorer performers (Reuter-Lorenz and Park, 2014).

In PDCN, strengthened recruitment of frontal PC_2_ (SMA couplings with executive/spatial areas) and posterior PC_1_ (precuneus couplings with executive areas) correlated with poorer working memory on the DOT, which engages executive control as digit strings of increasing length must be mentally reorganized into ascending order for recall. Thus, stronger integration of executive resources with both frontal and parietal regions may suggest diminished executive functioning. In contrast, better working memory in controls correlated with stronger expression of posterior PC_1_ and PC_6_ (occipitoparietal couplings with caudate/temporal pole) topologies, suggesting that integration of occipitoparietal resources with executive and semantic resources was beneficial. In PDCN, stronger posterior PC_1_ couplings also predicted poorer inhibitory control (CWINH), perhaps signifying frontal inefficiencies in suppressing prepotent responses, which are automatically activated in posterior cortex (Sebastian et al., 2013; Murray et al., 2017). Conversely, in controls stronger posterior PC_3_ coherence (inferior parietal couplings with spatial/memory hubs) was favorable for inhibition, consistent with parietal mediation of inhibition (Hampshire and Sharp, 2015), whereas stronger posterior PC_4_ connectivity (parieto-occipital couplings with precentral/cerebellar planning and inferior temporal semantic hubs) was detrimental, suggesting that integration of visuospatial and planning resources (Oshio et al., 2010; Stoodley et al., 2012; Cona et al., 2017) is costly for rapid inhibitory control. In PDCN, however, stronger posterior PC_4_ couplings predicted poorer phonemic fluency (LET), for which word retrieval is constrained by phonology. Phonological search in older adults is improved by segregation of frontal and semantic networks (Gonzalez-Burgos et al., 2021). By inference, in PDCN integration of parieto-occipital resources with semantic/executive resources may signify reduced efficiency in executive functioning.

Our results also aligned with the prominent effects of posterior cortex atrophy on spatial cognition in PD (Filoteo et al., 2014; Garcia-Diaz et al., 2018). Stronger posterior PC_1_ couplings correlated with poorer visuospatial processing (JLOT) in PDCN, suggesting dependence upon executive resources to analyze spatial details, possibly due to impoverished parietal processing of spatial content (Schott et al., 2019). Conversely, in controls poorer visuospatial processing correlated with stronger frontal PC_3_ connectivity (executive couplings with planning/spatial hubs), suggesting greater integration of diverse cognitive resources was needed to analyze spatial details in lower performers. Controls who expressed strengthened posterior PC_5_ couplings (inferior parietotemporal connectivity with executive hubs), a PDCN topology, also showed poorer visual organization on the HVOT, which tests naming of object drawings that are dismantled into puzzle-like pieces. Expression of this abnormal topology may therefore reflect difficulties organizing picture fragments into recognizable pictures in lower performers.

Mental rotation topologies also correlated with visuospatial memory on the BVMT where abstract drawings are reproduced for later recall. The BVMT emphasizes visual construction abilities needed to perceive object parts and reconstruct them from memory. Stronger SMA PC_2_ couplings with executive/spatial hubs correlated with poorer visuospatial memory in PDCN, as it did with working memory, perhaps signifying impoverished visual construction alongside diminished working memory, which supports episodic memory. Although PDCN expression of control topologies typically correlated negatively with cognition, an exception was that stronger frontal PC_3_ couplings (control feature) with spatial/planning hubs correlated with better visuospatial memory in PDCN, perhaps due to favorable influences of planning circuitry on visual construction. This interpretation assumes that frontal PC_3_ circuitry, including the right SMA, is sufficiently intact in higher performing patients to support efficient communications. Conversely, in controls strengthened frontal PC_3_ and occipitoparietal PC_2_ couplings with semantic (Ralph et al., 2017) and memory hubs were both unfavorable for visuospatial memory, possibly owing to diminished encoding alongside visual construction difficulties in poorer performers. Notably, stronger frontal PC_3_ expression also correlated with poorer visuospatial processing (JLOT) in the control group, underscoring the general neuronal processing resources of this topology for different facets of spatial cognition.

### Coupling topologies predict future cognitive progression

The expression of PDCN and control topologies had different prognostic implications for longitudinal decline. While PDCN topologies were unrelated to baseline cognition, stronger frontal PC_1_ and posterior PC_5_ expression typically protected against cognitive progression, indicating that functional reorganization was compensatory. Strengthened frontal PC_1_ couplings (preSMA, precentral, middle-frontal) with visuospatial hubs (angular gyrus, lingual gyrus) was a domain-general topology that predicted less decline or preservation of working memory, inhibitory control, and verbal episodic memory (CVLT). These results align with frontoparietal control of working memory and preSMA-parietal mediation of inhibitory control (Cai et al., 2012; Hampshire and Sharp, 2015). They are also compatible with frontal control of strategic word-list learning (e.g., categorization) for later recall, lingual gyrus recognition of words, and angular gyrus support of retrieval (Thakral et al., 2017). Less decline in verbal memory was also predicted by strengthened parietotemporal PC_5_ couplings with frontostriatal circuitry known to influence diverse cognitive functions including memory (Ekman et al., 2016). Altogether, functional reorganization provided alternative routes to handle visuospatial demands, which in turn sustained multiple cognitive functions longitudinally.

Conversely, expression of control group topologies predicted greater cognitive progression longitudinally, consistent with neuropathophysiological changes in these circuitries in PDCN at baseline. Greater longitudinal decline in phonemic fluency was predicted by strengthened inferior parietal PC_3_ connectivity with spatial/memory hubs and occipitoparietal PC_6_ connectivity with the caudate and temporal pole, a high-level convergence zone that governs semantic representations for all conceptual domains (Ralph et al., 2017). This finding reflects maladaptive influences of posterior cortical resources on phonemic fluency, for which more efficient phonemic searches are governed by frontal cortex (Tupak et al., 2012; Katzev et al., 2013).

Distinct topologies predicted longitudinal decline in two facets of spatial cognition that are risk factors for MCI and dementia conversion (Johnson and Galvin, 2011; Hong et al., 2014; Hobson and Meara, 2015; Chung et al., 2020). Greater decline in visuospatial memory (BVMT) was predicted by stronger precuneus PC_1_ couplings with executive hubs, indicating that integration of spatial and executive resources is a marker of visual construction disturbances. In contrast, greater decline in visual organization (HVOT) was predicted by stronger caudate/globus pallidus PC_4_ connectivity with the right parahippocampus. This finding can be understood by the projections that the striatum receives from the hippocampus, which in turn receives parietal and occipital-temporal inputs that carry visual information used in encoding objects and space (Kravitz et al., 2013). By inference, this result may signify reduced caudate selectivity of visual information (Hikosaka et al., 2019) alongside impoverished parahippocampus reactivation of details needed to recognize objects. Collectively, expression of these topologies foreshadowed future deterioration in spatial cognition.

Strengthened connectivity of two domain-general topologies, one a control feature (inferior parietal PC_3_ couplings with spatial/memory hubs) and another a PD feature (parietotemporal PC_5_ couplings with executive hubs), both predicted greater longitudinal decline in confrontation naming (BNT). Confrontation naming is a facet of semantic memory that requires generating names of pictures and is highly relevant to PD as it predicts conversion to dementia (Hobson and Meara, 2015). Whereas names for common objects are automatically retrieved, uncommon objects require cognitive resources to recognize objects and find their names (Hoffman et al., 2015). Thus, strengthened integration of parietal-temporal resources with spatial, memory, and executive resources may signify impoverished object representations (Harrington et al., 2021), which in turn may be a proxy for future dementia.

### Predictors of motor disability and progression

Although cognitive mechanisms underlying motor disability are not completely understood, motor symptoms in PD and cognitive functioning are not completely separable as cognition influences motor performance (Moustafa et al., 2016). For example, executive, attention and visuospatial decline are more severe in PD with FoG (Factor et al., 2014; Tard et al., 2015; Turner et al., 2021), and executive dysfunction affects gait timing, rhythmicity, and PIGD severity (Yogev-Seligmann et al., 2008; Kelly et al., 2015; Sosnik et al., 2022). Yet we found no correlations between baseline cognition and motor disability, likely owing to our PDCN cohort. Cognitive predictors of motor progression are also poorly understood, especially within short timeframes that would best inform intervention strategies. Recently, a study reported that better baseline visual memory and visuospatial ability predicted less risk for developing FoG within 2 years (Chung et al., 2021). Our study extends this finding by showing that declining visuospatial and visual construction over 2 years is prognostic of greater longitudinal motor and PIGD feature progression, suggesting deterioration in shared substrates. We also discovered that a link exists between mental transformations and real-life cognitive-motor disability as poorer hand-laterality accuracy was also prognostic of greater motor severity 2 years later, thereby signifying overlapping cognitive-motor resources.

Motor symptoms on the UPDRS are largely evaluated in active states, during movement, and therefore depend upon cognitive-motor control processes. This is true even for the performance of seemingly simple, repetitive movements where normally, the seamless intertwining of cognitive and motor functions controls the rhythm, amplitude, pacing and spatial aspects of motor sequences (Georgopoulos, 2002). When cognitive-motor control breaks down in PD it affects planning, online motor control, and coordination of simple movements (Harrington and Haaland, 1991; Snider et al., 2014). Our findings indicate that circuitries that respond to cognitive load during visuospatial processing, which supports goal-directed actions, are sensitive to the severity of motor disability and PIGD features, but not tremor. Neurodegenerative mechanisms of motor disability were elucidated by their correlations with control features, namely frontal (PC_2_) and posterior (PC_4_,_6_) topologies. While little attention has been paid to the mechanisms of motor or PIGD feature severity in task-activated fMRI studies, SMA dysfunction is common in PD during movement (Rowe et al., 2002; Wu et al., 2010; Herz et al., 2014) and mental imagery of gait in FoG (Snijders et al., 2011; Huang et al., 2021). We found that lower motor and PIGD feature severity at baseline both correlated with stronger SMA PC_2_ couplings with executive/spatial regions that normally support mental imagery and visuospatial/motor transformations (Cavanna and Trimble, 2006; Vilberg and Rugg, 2008; Ranganath and Ritchey, 2012; Schott et al., 2019). This finding aligns with the negative correlation between motor severity in PD and SMA activation during anti-phase movements (Wu et al., 2010). Thus, expression of this topology appears favorable for cognitive-motor control in patients without underlying neuropathophysiology. This prospect agrees with the SMA’s role alongside other executive and parietal regions in planning and adapting action plans to changing situations (e.g., narrow doorways, obstacles, turning) (Hughes et al., 2010; Snijders et al., 2011). However, frontal PC_2_ did not predict motor progression.

Rather, baseline and longitudinal motor and PIGD feature severity were, respectively distinguished by their correlations with posterior PC_4_ and PC_6_ topologies. The result aligns with the loss in the postural congruency effect for hand-laterality judgments after transcranial magnetic stimulation to the occipital-temporal but not the premotor cortex in PD (van Nuenen et al., 2012), indicating a shift in processing resources to posterior brain areas. Neither posterior topology predicted longitudinal changes in spatial cognition or other cognitive functions, suggesting an independent influence on motor progression. Stronger posterior PC_4_ expression (parieto-occipital couplings with planning/semantic hubs) predicted lower motor severity at baseline and longitudinally. This suggests that cognitive-motor control was favorably influenced by the integration of parieto-occipital resources, which represent spatial information and cognitive aspects of actions (Aflalo et al., 2015) with planning (Oshio et al., 2010; Stoodley et al., 2012; Cona et al., 2017) and semantic resources (Binder and Desai, 2011). Conversely, stronger posterior PC_6_ (calcarine cortex, superior occipital, precuneus) couplings with the caudate and temporal pole predicted greater longitudinal PIGD feature severity. Calcarine cortex connectivity was specifically increased with the right caudate, which selectively responds to visual stimuli (Esposito et al., 2021). This result may signify a shift to using visual information to control balance and locomotion, alongside recruitment of the temporal pole, which represents conceptual aspects of actions and the body (Ralph et al., 2017). This finding is also relevant to marked cholinergic reductions in the right caudate and temporal pole in PD with FoG (Bohnen et al., 2019), underscoring the vulnerability of these regions to cholinergic degeneration alongside parieto-occipital cortices (Bohnen et al., 2022). Altogether, distinct parieto-occipital coupling topologies have different prognostic implications for 2-year changes in motor and PIGD feature severity.

### Limitations

Testing patients on medications can mask functional abnormalities and affect cognition and motor disability. However, there are lingering effects of dopamine after overnight withdrawal. It is also important to understand neurobehavioral functioning in daily life when taking treatments. Second, inclusion of six *de novo* PDCN increases heterogeneity of the cohort. Despite this limitation, group differences in functional connectivity were robust. Coupling topologies were also sensitive to baseline and longitudinal changes in behavioral variables, partly owing to the improved temporal resolution of our multiband fMRI protocol (Tomasi et al., 2016). Third, neurocognitive correlations were medium in magnitude, likely due to restricted ranges on variables in PDCN, alongside the use of compensatory strategies, which could mask cognitive difficulties and mitigate correlations. Fourth, while longitudinal analyses controlled for aging, collection of longitudinal data in controls would better gauge rates of disease-related cognitive progression. Control group data would also help elucidate the meaning of improvements in some patients on various cognitive tasks, which could be related to test familiarity, despite using different test forms. For example, longitudinal studies of preclinical Huntington’s disease (Paulsen and Long, 2014) indicate that the influence of repeated testing on cognitive performances is related to disease burden. Healthy controls show linear improvements longitudinally on some cognitive tests whereas longitudinal changes in patients depend upon proximity to a manifest diagnosis. Whereas performances remain stable on the average in patients far from a manifest diagnosis, cognitive decline progressively accelerates as patients approach a manifest diagnosis. Of relevance to this issue is that in our study no participant who showed improved scores on one test also showed consistent longitudinal improvements on all cognitive tests. This aligns with the heterogeneous cognitive changes in PD but could also suggest that familiarity effects are not necessarily ubiquitous across all tests. A control group with longitudinal data would help sort out these issues. Lastly, although neurocognitive variables predicted longitudinal PIGD severity, quantitative measures of freezing should be considered (Ziegler et al., 2010) as the UPDRS may not be sufficiently sensitive to FoG, a facet of PIGD (Bluett et al., 2019).

## Conclusion

To summarize, markers of functional reconfiguration in response to increasing visuospatial demands were prognostic of longitudinal cognitive and motor progression. Stronger expression of control group topologies predicted greater cognitive progression, signifying emerging neuropathophysiology at baseline. Conversely, stronger expression of PDCN topologies predicted less decline or even preserved cognition longitudinally, demonstrating that functional reorganization provided alternative routes to handle increasing spatial demands, which in turn helped sustain interfacing cognitive functions. Distinct parieto-occipital topologies had different prognostic implications for longitudinal motor functioning, with expression of one topology protecting against motor decline and expression of another predicting greater PIGD feature severity. Concurrently, greater longitudinal decline in visuospatial and visual construction both portended greater motor and PIGD feature progression, suggesting deterioration in common resources. Collectively, these novel discoveries show that functional topologies underlying visuospatial cognition at baseline foreshadow future cognitive and motor progression.

## Data availability statement

The datasets presented in this article are not readily available because the data are not publicly available due to privacy or ethical restrictions imposed the United States Department of Veteran Affairs. The data that support the findings of this study are available on reasonable request from the corresponding author. Requests to access the datasets should be directed to DH, dharrington@ucsd.edu.

## Ethics statement

The studies involving human participants were reviewed and approved by the Institutional Review Board VA San Diego Healthcare System. The patients/participants provided their written informed consent to participate in this study.

## Author contributions

DH conceived and designed the study, performed statistical analyses, and wrote the first and final drafts of the manuscript. QS acquired the imaging data and performed the neuroimaging analyses. XW performed neuroimaging and statistical analyses and made substantive technical contributions. RL reviewed brain MRIs. IL, MH, XW, and RL contributed critical content to the manuscript revision. All authors read and approved the submitted version.

## Footnotes

1
http://afni.nimh.nih.gov

2
http://www.fmrib.ox.ac.uk/fsl/

3
http://surfer.nmr.mgh.harvard.edu
