# Supplementary material for: Functional topologies of spatial cognition predict cognitive and motor progression in Parkinson’s
Source: Front Aging Neurosci. 2022 Oct 10;14:987225. doi: 10.3389/fnagi.2022.987225 (PMC9589098; doi:10.3389/fnagi.2022.987225)
Supplement: Supplementary file 1 [file Data_Sheet_1.pdf]

SUPPLEMENTARY MATERIAL

MATERIALS AND METHODS

Analyses of Brain Atrophy

To determine if internetwork couplings in the PD group were related to brain atrophy, cortical thickness and volume were analyzed using FreeSurfer 5.3 (http://surfer.nmr.mgh.harvard.edu). Individual subject's cortical folding patterns were inflated, registered to a standard spherical surface template, and smoothed with 10mm FWHM Gaussian kernel to improve the signal-to-noise ratio and reduce local variations across subjects (Fjell et al., 2009). Tests of group differences in thickness and volume (age as a nuisance variable) were conducted across the continuous cortical surface using the FreeSurfer QDEC application and the false discovery rate (FDR, $q<0.05$) adjustment for multiple comparisons. Group differences were also tested for subcortical volumes of interest (bilateral caudate, putamen, and hippocampus), adjusting for total intracranial volume.

RESULTS

Hand-Laterality Judgements

All four angles were analyzed using a mixed-model ANOVA with group as the between subject factor and rotation angle (0°, 60°/300°, 120°/240°, and 180°) and hand as within-subject factors. The Huynh–Feldt correction was applied to multiple degree of freedom effects to adjust for violations of sphericity. Supplementary Figure 2 displays the results from this analysis. No group differences or group interactions with angle or hand were found for RT or percent correct. In both groups, rotation angle had robust effects on RT [$F(2.1, 215.9) = 392.9$, $p<.0001$, $\eta_p^2 = .80$] and percent correct [$F(1.5,192.6) = 139.8$, $p < .0001$, $\eta_p^2 = .57$]. There was no effect of hand or its interaction with angle on percent correct ($p > .24$). For RT, there was an hand by angle interaction ($F(2.1, 215.9)=4.7$, $p<.04$, $\eta_p^2 = .05$], which was due to the longer RT for the left than the right hand at all rotation angles ($p<.001$) except for 180° ($p>.10$). Supplementary Figure 2 shows that RTs were faster and accuracy was better for easier 0° and 60°/300° than for 120°/240°and 180° in support of grouping angles into easy and hard rotations. Altogether, these results show incremental and decremental effects of angel of disparity on RT and percent correct, respectively, consistent with the robust effects of easy versus hard angle contrast on brain activation (Figure 1B).

Upper Limb Motor Severity Effect on Hand Laterality Judgments

To determine if upper limb mmotor severity (UPDRS Part 3) influenced accuracy and RTs, separate ANOVA models for left and right-sided motor severity tested for its effects on percent correct and RT as a function of rotation angle (easy versus hard) and

hand. Right-sided upper limb severity and its interactions with within-subject factors were nonsignificant for RT and percent correct, as was left-sided upper limb severity for percent correct. For RT, there was an interaction of left-sided motor severity by angle by performing hand ($F(1,61)=9.5$, $p<.01$, $\eta_p^2 = .35$). Follow-up analyses indicated that for the left hand, greater left-sided motor severity was associated with longer RTs for easy ($r=.30$, $p<.016$), but not hard rotation angles ($p>.93$). For the right hand, left-sided motor severity had no effect on RTs regardless of rotation angle. Altogether, upper limb motor severity did not influence accuracy or RTs of hard rotation angles.

Group Differences in Brain Atrophy

Group differences in cortical thickness and volume were nonsignificant. Independent t tests (bias-corrected, bootstrapped) also failed to show group differences in left and right putamen, caudate, globus pallidus, and hippocampus volumes (FDR adjusted). Due to the lack of manifested atrophy in the PDCN group relative to controls, gray matter was not used as a covariate in subsequent analyses.

References

Fjell, A.M., Westlye, L.T., Amlien, I., Espeseth, T., Reinvang, I., Raz, N., et al. (2009). High consistency of regional cortical thinning in aging across multiple samples. *Cereb Cortex* 19(9), 2001-2012. doi: 10.1093/cercor/bhn232.

Supplementary Table 1. Seeds and MNI coordinates for gPPI analyses.

Seed	MNI
**Frontal**	
L superior frontal (BA 10)	-35 54 20
R middle frontal (BA 10)	45 51 0
R pSMA (BA 6)	6 26 46
L pSMA (BA 6)	-6 26 46
L inferior frontal (BA 45)	-44 26 28
R anterior insula (BA 13)	34 25 1
L anterior insula (BA 13)	-34 25 1
R SMA	9 11 49
L SMA	-9 11 49
B SMA (BA 6)	-1 6 60
L SMA (BA 6)	-12 2 67
R precentral (BA 6)	29 1 59
**Parietal**	
R supramarginal (BA 40)	49 -36 45
R inferior parietal (BA 4)	39 -44 39
R precuneus (BA 7)	14 -66 61
L precuneus (BA 7)	-20 -70 47
R angular gyrus (BA 39)	52 -78 11
**Occipital**	
R superior occipital (BA 19)	37 -82 31
L calcarine cortex	-10 -77 8
**Temporal**	
R inferior temporal (BA 37)	48 -64 -7
**Subcortical**	
L caudate	-12 8 10
R caudate	11 10 8
L putamen	-22 5 4
R putamen	21 7 2
L globus pallidus	-16 -1 1
R globus pallidus	17 -3 2

gPPI = generalized psychophysical interaction. SMA = supplementary motor area; pSMA = presupplementary motor area

Supplementary Table 2. Hard > easy angle-modulated couplings stronger in the control than the PDCN group.

Seed	Region (BA)	Voxels	MNI†	p value‡
**Frontal**	**Control > PDCN**			
L superior frontal (BA 10)	B precuneus (BA 7)	79	2 -75 42	4.00E-05
	R putamen	43	32 0 -7	7.00E-06
R middle frontal (BA 10)	R putamen	38	28 -6 -5	7.00E-06
L inferior frontal (BA 45)	L fusiform	92	-36 -48 -19	3.4098E-07
	R superior temporal	71	66 -18 12	2.70E-05
R insula (BA 13)	L precentral (BA 6)	182	-57 4 11	2.2465E-07
	R putamen	153	33 0 -8	6.5856E-10
L insula (BA 13)	R putamen	41	23 6 5	1.00E-06
R SMA (BA 6)	L precentral (BA 6)	82	-52 3 7	2.00E-06
L SMA (BA 6)	B medial frontal (BA 10)	118	-3 63 17	1.94E-04
	L anterior cingulate (BA 32)	77	-1 43 13	4.00E-05
B SMA (BA 6)	L precuneus	167	-9 -71 38	4.00E-06
L SMA (BA 6)	R precuneus	84	14 -64 34	5.00E-06
**Parietal**				
R inferior parietal (BA 4)	L precuneus	124	-5 -40 55	3.00E-06
	R precuneus	78	8 -67 22	9.00E-06
	R parahippocampus	33	27 -27 -15	1.40E-05
R precuneus (BA 7)	R tonsil lobule VIII	76	39 -46 -48	8.8165E-07
L precuneus (BA 7)	R medial superior frontal (BA 9)	160	4 51 20	1.30E-05
	L medial frontal (BA 11)	135	-1 59 -20	8.4521E-09
	R medial frontal (BA 10)	131	8 63 11	6.90E-05
	R posterior cingulate	99	1 -59 25	4.70E-05
	L middle temporal pole	74	-61 9 -28	9.00E-06
R angular gyrus (BA 39)	L parahippocampus	33	-19 -39 0	5.2192E-07
**Occipital**				
R superior occipital (BA 19)	L middle temporal pole	108	-61 6 -14	9.00E-06
	L globus pallidus	68	-21 -11 -8	4.166E-08
	L parahippocampus	38	-17 -39 -9	1.10E-05
L calcarine cortex	L anterior middle temporal (BA 21)	113	-66 0 -15	9.9951E-08
	R caudate	36	16 2 13	2.1138E-07
**Subcortical**				
L caudate	L parahippocampus	47	-25 -16 -11	3.00E-06
	R parahippocampus	44	25 -28 -10	9.00E-06
L putamen	R putamen	33	-22 13 6	1.30E-05
R globus pallidus	L tonsil lobule VIIIa	177	-29 -48 -57	3.00E-06
	R middle frontal (BA 10)	170	39 55 -9	2.00E-05
	R cuneus	98	5 -82 27	9.00E-06
	L lingual	73	-9 -101 -4	9.20E-05
	R superior middle (BA 40)	72	59 -24 27	2.20E-05
	R parahippocampus	48	28 -5 -13	5.00E-06

†Montreal Neurological Institute (MNI) brain atlas coordinates.
‡ Tabled p values are uncorrected, but all values remained significant after FDR adjustment ($p < .001$) for 53 PPI features that showed group differences (Supplementary Tables 3 and 4). BA = Brodmann area; SMA = supplementary motor area

Supplementary Table 3. Stronger angle-modulated (hard > easy) couplings in the PDCN than the control group.

Seed	Region (BA)	Voxels	MNI†	p value‡
**Frontal**	**PDCN > Control**			
R middle frontal (BA 10)	L angular gyrus (BA 39)	77	-53 -72 27	4.00E-06
R preSMA (BA 6)	L angular gyrus (BA 39)	117	-50 -73 28	3.00E-06
L preSMA (BA 6)	L angular gyrus (BA 39)	276	-49 -75 32	3.00E-06
	R angular gyrus (BA 39)	147	52 -72 35	2.00E-06
R SMA (BA 6)	L middle occipital	72	-32 -79 7	1.8655E-07
R precentral (BA 6)	L lingual	74	-20 -82 -7	1.70E-05
**Parietal**				
R SMG (BA 40)	R middle frontal (BA 46)	84	49 45 12	6.00E-06
	R caudate	45	18 15 17	8.38350E-07
R angular gyrus (BA 39)	L precentral	95	-46 -10 42	1.00E-06
**Occipital**				
R superior occipital (BA 19)	R inferior temporal	185	48 -15 -31	6.6957E-11
**Temporal**				
R inferior temporal (BA 37)	B thalamus medial dorsal	113	-1 -11 18	1.9777E-08
**Subcortical**				
L putamen	L precuneus	232	-17 -62 53	1.70E-05
L globus pallidus	L precuneus/superior parietal (BA 7)	188	-17 -59 52	9.00E-06
	L inferior frontal (BA 46)	152	-50 25 14	2.20E-05
	L posterior insula	102	-35 -1 16	3.877E-09

†Montreal Neurological Institute (MNI) brain atlas coordinates.

‡ Tabled p values are uncorrected, but remained significant after FDR adjustment ($p < .001$) for 53 PPI features that showed group differences (Supplementary Tables 3 and 4). BA = Brodmann area; preSMA = presupplementary motor area; SMA = supplementary motor area; SMG = supramarginal gyrus

Supplementary Table 4. Hard > easy couplings that did not differ between the PDCN and control groups.

Seed	Region (BA)	Voxels	MNI†	p value‡
**Frontal**				
L superior frontal (BA 10)	R postcentral (BA 5)	75	28 -35 69	5.4246E-04
R middle frontal (BA 10)	L middle occipital (BA 18)	444	-13 -103 15	7.5193E-05
	R middle occipital (BA 19)	392	36 -87 21	2.2608E-04
	R paracentral (BA 5)	347	2 -29 52	3.1648E-06
	L lingual (BA 18)	233	-9 -72 7	1.3365E-05
	B SMA (BA 6)	224	-2 -2 56	1.2602E-04
	L superior frontal (BA 9)	202	-24 53 30	5.6532E-05
	R precentral (BA 4, 6)	190	40 -11 47	7.5620E-06
	R superior temporal (BA 22)	179	64 -13 4	8.5529E-05
	L cuneus (BA 18)	168	-13 -73 29	4.2429E-05
	L putamen	134	-19 7 14	2.9216E-07
	L putamen	131	-25 8 -6	2.6354E-06
	R superior temporal (BA 22)	125	49 -20 8	8.0378E-07
	L medial frontal (BA 10)	104	-1 63 -9	1.1529E-04
	R precuneus (BA 7)	104	26 -44 50	6.4914E-07
	L insula (BA 13)	94	-45 -1 -6	1.8090E-05
	R thalamus	89	9 -15 7	9.8328E-07
	L medial frontal, anterior cingulate (BA 9, 32)	86	-1 39 24	3.3142E-04
	L middle frontal (BA 8)	83	-29 21 45	7.3707E-05
	R putamen	78	24 -1 2	2.8370E-06
	L precuneus (BA 7)	77	-11 -45 44	5.5413E-06
	L fusiform (BA 37)	74	-41 -51 -19	1.2579E-04
	L parahippocampus (BA 28)	72	-21 -14 -8	2.7875E-05
	R putamen	63	26 18 -3	5.3072E-05
	R caudate	62	15 11 -8	7.5218E-06
R PreSMA (BA 6)	R putamen	205	22 10 -1	2.6364E-07
L preSMA (BA 6)	R putamen	60	20 10 -5	1.1506E-04
L inferior frontal (BA 45)	L superior temporal (BA 38)	107	-46 15 -21	9.0779E-07
R insula (BA 13)	L putamen	345	-25 11 -1	1.1099E-08
	R pre/postcentral (BA 4,3)	203	32 -24 62	1.4017E-05
	L posterior cingulate (BA 31)	140	-4 -58 20	7.3970E-05
	L fusiform (BA 37)	98	-35 -42 -15	4.9348E-06
L insula (BA 13)	L superior temporal (BA 38)	154	-53 11 -23	1.5265E-05
	R putamen	131	21 15 -4	1.4901E-05
	R superior temporal (BA 38)	106	44 19 -37	5.4300E-05
R SMA (BA 6)	R putamen	105	23 13 -5	1.4094E-04
	R pre/postcentral (BA 3,4)	96	37 -17 51	2.6326E-04
	L putamen	95	-27 11 1	1.6885E-05
L SMA (BA 6)	R putamen	91	25 13 -7	4.3580E-05
	L inferior temporal (BA 20)	89	-59 -15 -35	6.3304E-06
	R middle temporal (BA 21)	70	63 -9 -15	3.6286E-04
	L putamen	37	-23 13 -9	8.2673E-04

B SMA (BA 6)	B medial frontal (BA 10)	1538	-1 56 12	2.7923E-05
	R middle temporal (BA 22)	198	59 -39 5	1.7528E-04
	R putamen	145	23 13 3	1.2555E-05
	L middle temporal (BA 21)	128	-60 -9 -11	8.3632E-05
	L superior temporal (BA 38)	98	-49 15 -23	2.2623E-05
	R middle temporal (BA 21)	83	54 1 -23	1.6428E-04
	L putamen	60	-26 12 -1	4.3989E-04
	L caudate body	37	-18 16 11	5.5114E-05
	R parahippocampus (BA 37)	35	25 -49 -8	2.5068E-04
R precentral (BA 6)	R postcentral (BA 3)	293	43 -19 56	2.8435E-05
	R precentral (BA 6)	160	22 -12 72	1.6787E-05
**Parietal**				
R SMG (BA 40)	L posterior cingulate (BA	292	0 -60 26	4.4104E-06
	23)	274	0 60 -15	3.4426E-06
	B medial frontal (BA 10,11)	103	21 14 1	4.0616E-06
	R putamen	99	34 -19 54	1.3077E-05
	R pre/postcentral (BA 4,3)	79	-23 12 5	1.4350E-07
	L putamen	47	-20 -55 5	1.6538E-04
	L lingual gyrus (BA 18)	43	-28 -20 -23	2.8376E-05
	L parahippocampus (BA 36)			
R inferior parietal (BA 40)	B medial frontal (BA 10)	192	0 59 -13	7.65545E-05
R precuneus (BA 7)	L middle temporal (BA 21)	93	-55 -10 -17	6.72847E-06
	L putamen	54	-23 12 -2	1.59416E-04
L precuneus (BA 7)	L fusiform (BA 37)	132	-22 -84 -10	1.62497E-05
R angular gyrus (BA 39)	R putamen	77	19 16 6	1.26080E-05
	L putamen	38	-23 13 -4	3.25598E-04
**Occipital**				
R superior occipital (BA 19)	L medial frontal (BA 10)	477	-3 62 -5	2.12920E-05
	L putamen	413	-22 10 3	5.63414E-08
	R putamen	325	21 15 2	1.38819E-07
	B posterior cingulate (BA	234	-1 -58 27	3.08525E-04
	31)	179	41 -25 2	1.70450E-08
	R insula (BA 13)	84	-63 -13 -9	3.80588E-05
	L middle temporal (BA 21)	84	-5 -17 63	3.12333E-04
	L SMA (BA 6)	79	-47 -4 -24	7.57115E-06
	L middle temporal (BA 38)	64	22 -45 2	3.84989E-05
	R parahippocampus (BA 30)			
L calcarine cortex	L putamen	71	-26 5 -7	5.70350E-06
	R putamen	46	24 6 -6	1.89495E-04
	R parahippocampus (BA 28)	43	19 -6 -13	3.26242E-05
**Temporal**				
R inferior temporal (BA 37)	R middle temporal (BA 21)	91	54 -4 -23	1.13127E-05
	L cingulate (BA 31)	70	-6 -45 30	3.45866E-05
**Basal Ganglia Seeds**				
L caudate	R putamen	58	19 9 7	1.14359E-04
	L putamen	34	-31 5 3	3.99374E-04
R caudate	R putamen	121	23 13 1	1.13063E-05
R globus pallidus	L pyramis	78	-21 -85 -33	3.17370E-04

‡ Tabled p values are uncorrected, but were significant after FDR adjustment ($p < .001$) for for 85 PPI features that showed angle-modualted effects (hard > easy) in both groups.
†Montreal Neurological Institute (MNI) brain atlas coordinates.
BA = Brodmann area; preSMA = presupplementary motor area; SMA = supplementary motor area; SMG = supramarginal gyrus

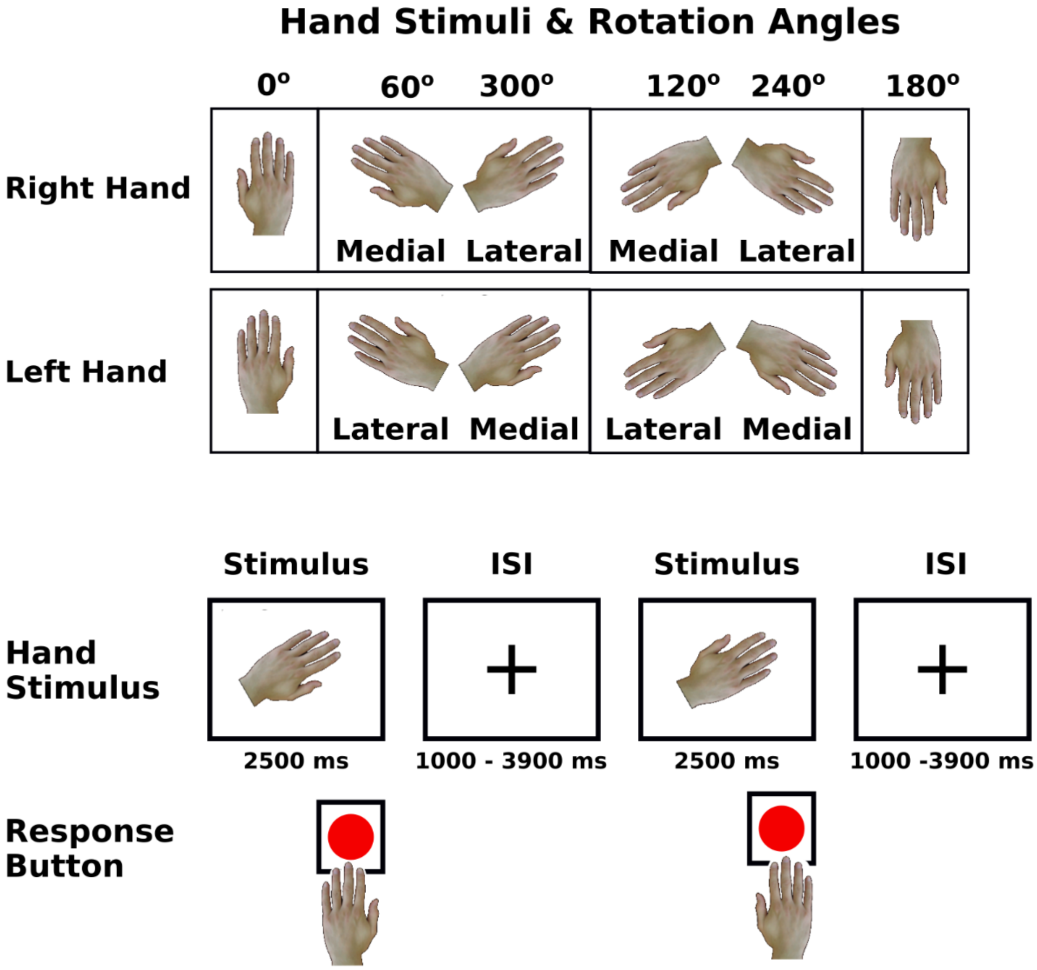

Supplementary Figure 1. Illustration of stimuli for the hand-laterality task and the trial events. The top figure displays hand stimuli for each of the rotation angles, which were oriented at 0°, 60°/300°, 120°/240°, and 180° with respect to the sagittal plane of the body when subjects laid supine in the scanner with their arms at their sides, hands flat with palms down, and fingers pointing straight in the direction of the feet (i.e., 0° with respect to body). The bottom figure illustrates the trial events. On each trial a picture of a hand was presented for 2500 ms and the subject responded as quickly as possible, judging whether the picuture was a left or right hand by making a left or right index finger keypress. Intertrial intervals consisted of randomly jittered (1000 ms to 3900 ms) filler trials where the participant fixated on a central crosshair.

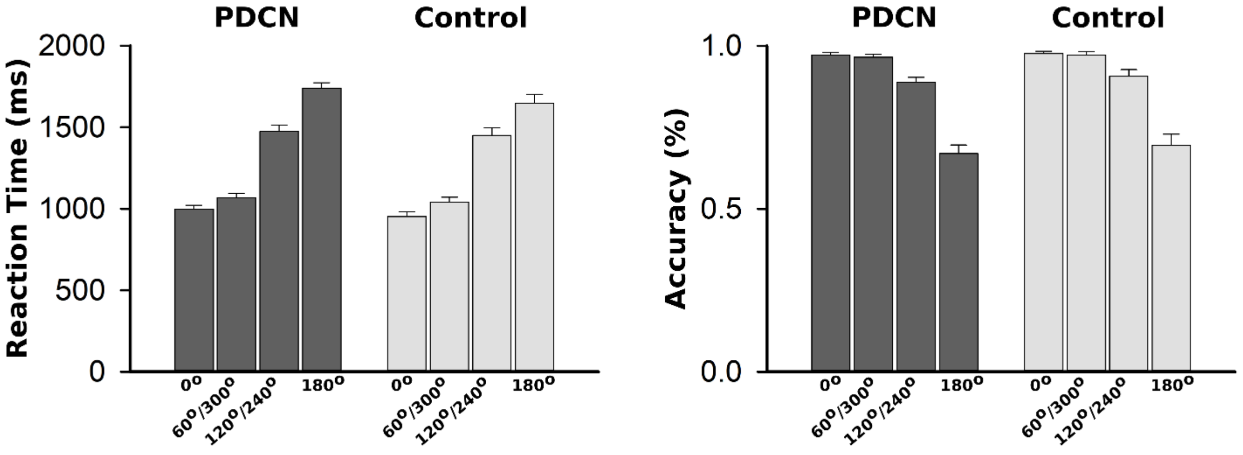

Supplementary Figure 2. Roation angle effects on hand laterality task performance in the PDCN and Control groups. Group means and standard errors are plotted for reaction time and accuracy as a function of rotation angle (0°, 60°/300°, 120°/240°, and 180°).

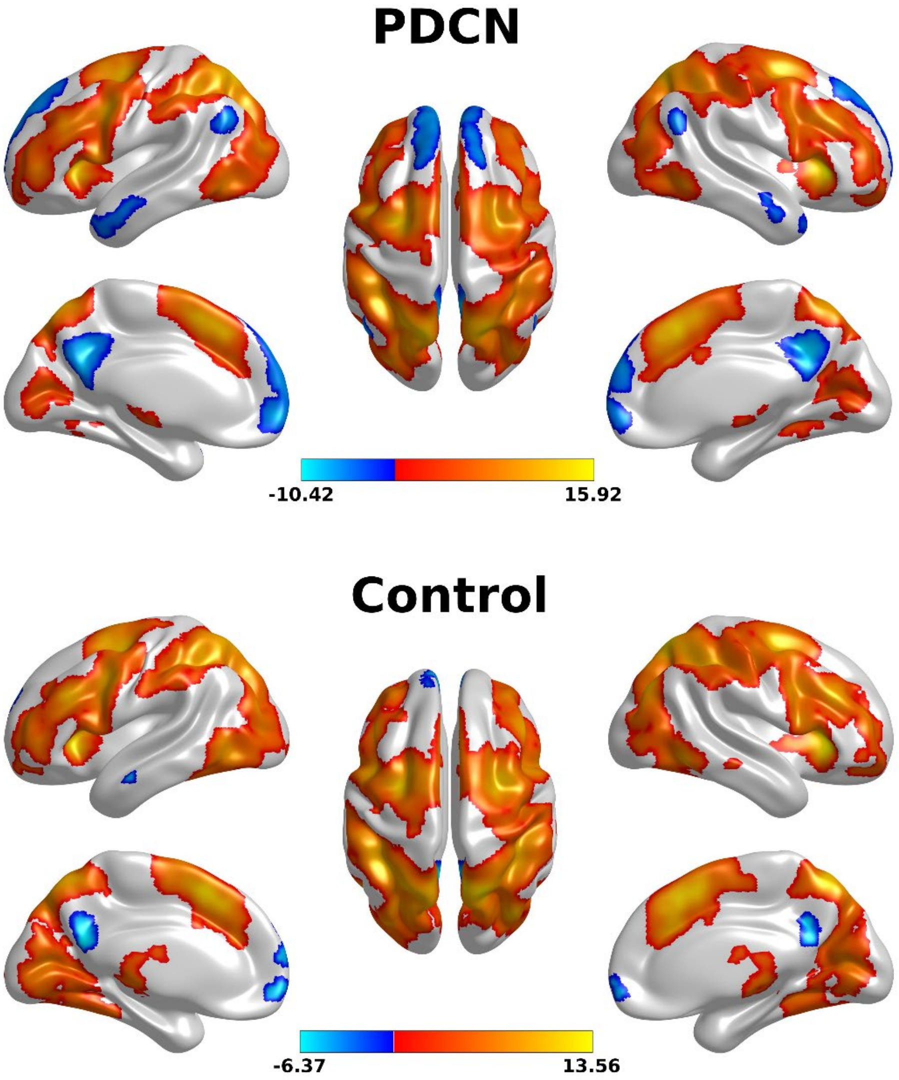

Supplementary Figure 3. Rotation angle effects on brain activation in the PDCN and control groups. The figure display left and right hemisphere regional activations from voxelwise tests of rotation angle effects in each group. Warm colors designate activations that were greater for larger (hard) than smaller (easy) angles of disparity. Cool colors designate activations that were greater for easy than hard angles of disparity. The color bar shows the range of F values for significant angle effects, were tested using a voxelwise probability of $p < .0001$ and a minimum cluster size of 19 voxels to obtain a familywise $p < .05$. Voxelwise tests for the group and group by rotation angle interaction effects were nonsignificant (i.e., familywise $p > .05$ using a voxelwise of $p < .001$ and a minimum cluster size of 50 voxels).

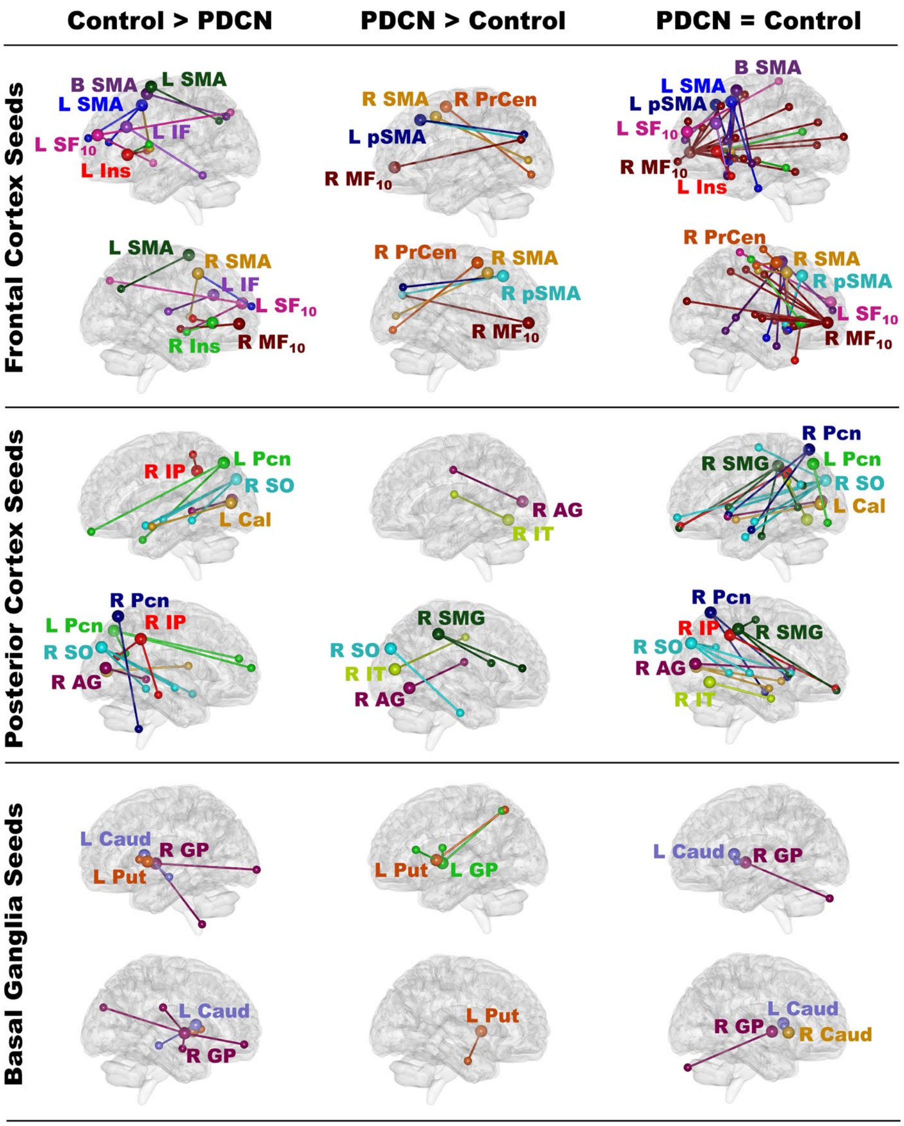

Supplementary Figure 4. Group differences in angle-modulated functional connectivity from generalized psychophysical interaction (gPPI) analyses. The figure illustrates the connections of a seed ROI (large balls) with other brain regions (small balls) that were stronger for hard than easy angles. Seeds and their connections are color coded and separated into frontal including anterior insula, posterior cortical, and basal ganglia seeds. In one or both groups, hard angle seed time-courses correlated more positively with the time courses of other brain voxels than easy angle seed time-courses. Columns display seed connections that were stronger in the control (Control > PDCN) or the PDCN group (PDCN > Control). The right column shows seed connections for which the strength of angle-modulated connectivity did not differ between groups (PDCN = Control). Supplementary Tables 2, 3, and 4 detail the volumes and spatial coordinates of connectivity features, and the p values for tests of group differences in connectivity features. L = left hemisphere; R = right hemisphere; Brodmann areas (BA) for frontal regions are designated by subscripts. AG = angular gyrus;Cal = calcrine cortex; Caud = caudate; GP = globus pallidus; IF = inferior frontal; Ins = insula; IP = inferior parietal; IT = inferior temporal; MF = middle frontal; Pcn = precuneus; PrCen=precentral; Put = putamen; preSMA = presupplementary motor area; SF = superior frontal; SMA = supplementary motor area; SMG = supramarginal gyrus; SO= superior occipital

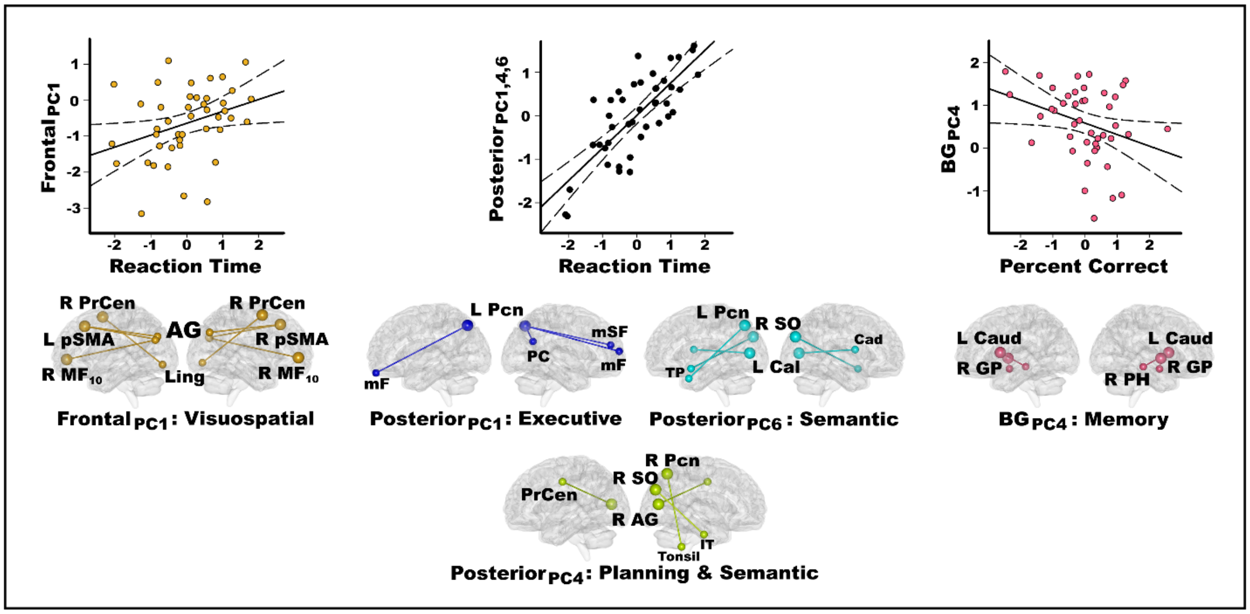

Supplementary Figure 5. Principal component score correlations with hand laterality performance in the control group. Plots display the best-fitting linear regression line (solid line) and 95% conference intervals (dotted lines) for significant correlations between age-adjusted reaction time (RT) and percent correct (x axis) and PC connectivity topologies (y axis). For each PC, seed(s) (large balls) and their connection(s) (small balls) are color coded. More positive RT values reflect slower responses for hard than easy rotations. More negative percent correct values reflect lower accuracy for hard than easy rotation angles. For RT (middle plot), predicted values from the regression equation are plotted for posterior PC_1, PC_4, and PC_6 [$\sum$ intercept + ($beta_{PC1}$ * PC_1 score) + ($beta_{PC4}$ * PC_4 score) + ($beta_{PC6}$ * PC_6 score)] = [$\sum$-.78 + (.30 * PC_1 score) + (.61 * PC_4 score) + (.40 * PC_6 score)]. B) PC correlations with motor symptom severity (Motor) and postural instability gait disorder severity (PIGD) at the baseline study visit (V1).

Brodmann areas for frontal seeds are designated by subscripts. L = left hemisphere; R = right hemisphere. AG = angular gyrus; Cad = caudate; Cal = calcarine cortex; GP = globus pallidus; IT = inferior temporal; MF = middle frontal; mF = medial frontal; mSF = medial superior frontal; Pcn = precuneus; PC = posterior cingulate; PrCen = precentral; pSmA = pre-supplementary motor area; SMA = supplementary motor area; SO = superior occipital; TP = temporal pole
